# Integrated analysis of mRNA and protein expression profiling in tubal endometriosis

**DOI:** 10.1530/REP-19-0587

**Published:** 2020-03-02

**Authors:** Hang Qi, Huiyu Zhang, Xiaoya Zhao, Ya Qin, Guiling Liang, Xiaoqing He, Jian Zhang

**Affiliations:** 1 Department of Obstetrics and Gynecology, International Peace Maternity and Child Health Hospital, School of Medicine, Shanghai Jiaotong University, Shanghai, China; 2 Shanghai Key Laboratory Embryo Original Diseases, Shanghai, China; 3 School of Life Science and Technology, ShanghaiTech University, Shanghai, China

## Abstract

Tubal endometriosis (tubal EM) is a subtype of endometriosis (EM) associated with fallopian tube impairments and infertility. Since the molecular mechanism underlying tubal EM is not clear, we assume that an aberrant transcriptome of fallopian tube epithelium and microenvironment changes caused by cytokines in tubal fluid are possible causes. The aim of this study was to identify potential hub mRNAs/proteins of tubal EM through integrated transcriptomic and proteomic analyses and to elucidate significant pathways, cellular functions, and interaction networks during the initiation and progression of tubal EM. We obtained human fallopian tube epithelium and tubal fluid samples from patients with and without tubal EM. Tubal epithelia were analyzed using microarray, and tubal fluid was analyzed using quantitative label-free LC-MS/MS. We identified differentially expressed genes (DEGs) and differentially expressed proteins (DEPs) and determined common mRNAs/protein. We observed 35 commonly deregulated mRNAs/proteins, and IPA indicated that cellular movement, inflammatory response, and immune cell trafficking were significantly activated during the pathogenesis of tubal EM. We also identified acute phase response signaling pathway activation as a unique pathogenesis signature of tubal EM. Our results demonstrate that an integrated analysis of the transcriptome and proteome has the potential to reveal novel disease mechanisms at a molecular level.

## Introduction

Fallopian tube epithelium contains large numbers of ciliated and secretory epithelial cells. Fallopian tube fluid is a complex mixture of components secreted from the epithelial cells and transudate fluid from blood plasma (Leese 1988). Fine synergistic regulation between fallopian tube epithelial cells and tubal fluid maintains stability of the fallopian tube microenvironment, promotes the embryo and fallopian tube interaction, and is a necessary prerequisite for successful fertilization, early embryo development, and embryo transport (Maillo *et al*. 2015, Li & Winuthayanon 2017). Thus, the fallopian tube plays an important role in establishing successful pregnancy.

Endometriosis (EM) is a chronic inflammatory disease associated with pain and infertility. Although the prevalence of this disease is likely underestimated, approximately 7% to 15% of women of reproductive age (Sayasneh *et al*. 2011) and 52.7% of women between 18–29 years old at the time of diagnosis are thought to be affected (Fuldeore & Soliman 2017). EM is a frequent contributor to infertility, either alone or in combination with other factors, such as peritoneal inflammation (Tanbo & Fedorcsak 2017).

Tubal EM is a subtype of pelvic EM and is characterized by the proliferation of functional endometrial glands and stroma in the fallopian tube. Endometrial implants are present in the fallopian tubes in 60% (21/35) of EM patients (Xia *et al*. 2018, Qi *et al*. 2019). Repeated cycles of hemorrhage result in fibrosis, scarring, and distention of the fallopian tube with blood, a condition known as hematosalpinx (Rezvani & Shaaban 2011). Moreover, patients with tubal EM exhibited lower ciliary beat frequency and weaker muscular contractile activity (Xia *et al*. 2018). Thus, tubal EM may cause infertility in women by affecting fallopian tube function (Xia *et al*. 2018). However, the pathogenesis of tubal EM-associated infertility is unclear.

Though global patterns of protein expression are analogous to that of mRNA transcript synthesis, discrepancies between transcript and protein product contribute to posttranscriptional regulatory mechanisms in cellular development (Tian *et al*. 2004). mRNA microarray detects only the changes at the mRNA level, which differs from the protein level. Therefore, it is crucial to integrate the transcriptome and proteome to explore the underlying mechanism of tubal EM.

Over the last decade, through the use of microarray analysis, investigators have reported differential gene expression in the eutopic endometrium of women with and without EM (Sherwin *et al*. 2008). Also, several studies have examined the proteome of eutopic endometrium (Kyama *et al*. 2006, Fassbender *et al*. 2012), peritoneal fluid (Williams *et al*. 2015), urine (El-Kasti *et al*. 2011, Williams *et al*. 2015), and menstrual blood (Hwang *et al*. 2013) in women with EM. However, profiles of differentially expressed genes (DEGs) and differentially expressed proteins (DEPs) in tubal EM are inconsistent, and the interaction between fallopian tube epithelium and tubal fluid has received relatively little attention. In the current study, we profiled DEGs in the tubal epithelium and DEPs in the tubal fluid from women with tubal EM and compared these to control samples to investigate similarities between DEGs and DEPs. Our results reveal dysregulation of specific pathways involved in acute phase response signaling in tubal EM samples. Together, our integrative analysis provides new insights into the pathogenesis of tubal EM and associated infertility.

## Materials and methods

### Patients and sample collection

This study was approved by the institutional ethics committee of the International Peace Maternity and Child Health Hospital in Shanghai, China (No. GKLW 2015-34). Written informed consent was obtained from each participant before enrolment.

Tubal epithelium and fallopian tube fluid specimens were collected from four women with tubal EM (case group) and four women with uterine leiomyoma without pelvic EM (control group) at the Department of Obstetrics and Gynecology of the International Peace Maternity and Child Health Hospital, Shanghai, between June 2016 and August 2017. All diagnoses dependent on histopathological examinations and tubal EM were confirmed by at least two experienced pathologists.

Tubal epithelium tissues were obtained from resected fallopian tubes, and fallopian tube fluid from the same participants was collected during gynecological surgery. Before collecting samples, we put PBS at 4°C for 1 h in hospital operating room refrigerator to precool PBS. The removed tubal epithelium tissues were immediately washed two times in 20 mL sterile pre-cooling PBS to remove blood. Fallopian tubal fluid was collected by flushing the lumen from isthmus part to fimbriae part of the dissected fallopian tube with 2 mL sterile precooled PBS (Yu *et al*. 2016).The tubal epithelium tissues were snap frozen in liquid nitrogen, and tubal fluid was placed on ice before transporting to the laboratory within 10 min. Then, tubal fluid was centrifuged at 300 ***g*** for 10 min at 4°C to remove cellular debris. After centrifugation, the pellet was removed and supernatant was stored at −80°C until extraction. Patient demographics and clinical pathological data were also collected.

### Microarray analysis

Total RNA was extracted from fallopian tube tissue samples using TRIzol reagent (Invitrogen) and purified using the RNeasy Mini kit (Cat. #74106, QIAGEN GmBH, Germany) following the manufacturer’s instructions. Then, the RNA preparations were checked for a RIN number to inspect RNA integration by an Agilent Bioanalyzer 2100 (Agilent technologies). The RINs for the samples and the concentration of RNA used for hybridization are listed in Supplementary Table 1 (see section on supplementary materials given at the end of this article). Sample preparation, hybridization, microarray wash and scanning, and feature extraction were carried out using the Agilent One-Color Microarray-Based Gene Expression Analysis (Low Input Quick Amp Labeling) protocol, version 6.3 (Agilent Technologies) with minor modifications. In brief, a random priming technique (Low Input Quick Amp Labeling Kit, One-Color) was used to amplify and transcribe each sample into fluorescent cRNA without a 3′ bias. The RNeasy Mini Kit (QIAGEN) was utilized to refine fluorescent cRNAs, and a NanoDrop ND-1000 UV-VIS spectrophotometer (NanoDrop) was used to calculate the specific activity and concentration. To fragment each labeled cRNA, we added 1 μL of 25× fragmentation buffer and 5 μL of 10× blocking agent in 1 μL to each labeled cRNA. The solution was then incubated for 30 min at 60°C and diluted in 25 μL of 2 × GE × Hybridization Buffer HI-RPM. Finally, 50 μL of hybridization solution was applied to the gasket slide and dispensed into the mRNA expression microarray slide. Slides were then kept in a hybridization oven (Agilent) for 17 h at 65°C. Slides were scanned by Agilent Microarray Scanner (Cat#G2565CA, Agilent technologies) with default settings, dye channel: green, scan resolution = 3 μm, PMT 100%, and 20 bit. Data were extracted with Feature Extraction software 10.7 (Agilent technologies). Raw data were normalized by Quantile algorithm, limma packages in R.

### Quantitative label-free LC-MS/MS and protein identification

The total volume of each sample was centrifuged at 14,000 ***g*** for 15 min. In total, 500 mL of supernatant was transfered into a 10-kDa ultrafiltration centrifuge tube and centrifuged at 12,000 ***g*** for 30 min. After centrifugation to a volume less than 100 μL, the remaining samples were added again until all samples were ultrafiltrated. The filtrate was discarded. Protein extracts from fallopian tube fluid were digested with trypsin according to the filter-aided sample preparation (FASP) procedure (Wisniewski *et al*. 2009). Mass spectrometric analysis of peptides was carried out on a QExactive Orbitrap mass spectrometer (Thermo Fisher Scientific). The mass spectrometer was operated in full-scan high resolution and profile mode. All mass spectra were acquired in positive ion mode and the column size was 0.075 mm×150 mm (ReproSil-Pur 120A C18-AQ 3.0 μm, Dr Maisch GmbH, Ammerbuch, Germany). The automatic gain control (AGC) target for MS1 acquisition was set to 1.0 × 10^6^ with a maximum ion injection time of 50 ms, and MS2 acquisition was 1.0 × 10^5^ with a maximum ion injection time of 80 ms. The dynamic exclusion duration was set to 30 s. MS1 survey scans (m/z 350–1800) were acquired with a resolving power of 70,000 at m/z 200. The MS2 Activation Type was higher energy collision-induced dissociation (HCD). Resolution for HCD spectra was set to 17,500 at m/z 200. The isolation window for the MS/MS was 2 Da, and the normalized collision energy was 30 eV. The instrument was operated in an enabled peptide recognition mode. The LC-MS/MS experiments were performed in triplicate for each sample.

### Protein–-mRNA correlation analysis

The interaction of microarray detected mRNAs and LC-MS/MS detected proteins were found according to the common official gene symbols from HGNC (HUGO Gene Nomenclature Committee) (https://www.genenames.org/, Last updated: 2020-02-22). Then, differential gene expression levels between patients with tubal EM and healthy controls were estimated with a moderated *t*-test using the limma package in R. *P*-values were corrected using the false discovery rate (FDR). Genes were considered differentially expressed if the adjusted *P* value was 0.01 and absolute fold-change was 2. In addition, raw MS data were analyzed using MaxQuant software version 1.6.0.16 (Max Planck Institute of Biochemistry in Martinsried, Germany). Protein identification was achieved by searching the MS/MS spectra against the NCBI UniProt Homo sapiens database containing 92,607 protein sequences (fasta file downloaded July 2016). Identification parameters were set as follows: fixed modifications: carbamidomethyl(C) and variable modifications: Oxidation (M) and Acetyl (Protein N-term). The false discovery rate (FDR) of protein and peptide identification was set to <0.01. We then analyzed DEGs and DEPs in tubal EM patients and compared these to the control group, with the data represented by a volcano plot (generated using the ggplot2 R package) and heatmap (generated using the pheatmap R package). To analyze the correlation between the transcriptome and proteome, changes in protein levels correlating with changes in the corresponding transcripts were investigated, and the overlap between DEGs and DEPs was demonstrated with Venn Diagrams (generated using the VennDiagram R package) and heatmap. A fold change >2 and *P *< 0.01 were set as cut-off criteria.

### Ingenuity pathway analyses of DEGs, DEPs, and common deregulated mRNAs/proteins

Identified DEGs, DEPs, and common differentially expressed mRNAs/proteins in the tubal EM, containing gene identifiers and corresponding expression values, were uploaded to the IPA software (QIAGEN). The ‘core analysis’ function included in the software was used to interpret the differentially expressed data, which included the identification of biological processes, canonical pathways, upstream transcriptional regulators, and gene networks. Each gene identifier was mapped to its corresponding gene object in the Ingenuity Pathway Knowledge Base (IPKB, Genes + Endogenous Chemicals). Default settings were used to determine the activation state of canonical pathways and upstream regulators using the global molecular network contained in the IPA knowledge base. Changed analysis settings were: interactions = direct and indirect and confidence = experimentally observed.

### Confirmation of DEGs by qRT-PCR and conformation of DEPs by ELISA

Ten out of all differentially expressed mRNAs were selected for validation by qPCR, including eight up-regulated genes (*IL6*, *TNFA*, *C2*, *C4B*, *CP*, *HP*, *SAA4*, and* ORM2*) and two down-regulated genes (*AHSG* and *MAP2K6*). To further verify expression changes in genes associated with tubal EM, fallopian tube epithelium tissues from four patients with tubal EM and four participants without tubal EM were transferred to the laboratory immediately after adding liquid nitrogen, and total RNA was extracted with Trizol Reagent (Invitrogen). One microgram of RNA was reverse-transcribed using a Revert-Aid First Strand cDNA Synthesis Kit (Takara). cDNA was then used as template for each PCR reaction using QuantiNova SYBR Green PCR Kit (QIAGEN) and the QuantStudio 7 Flex Real-Time PCR system (ThermoFisher). To determine relative gene expression, *GAPDH*, *ACTB*, and *18s RNA* was utilized as an internal control, and the expression level of ten genes were standardized to *GAPDH*, *ACTB*, and *18s RNA*. Primers specific to DEGs were designed online using the primer design program from Sangon Biotech (Shanghai) Co., Ltd. (http://www.sangon.com/primerDesign) and normalizer gene primers were used as reported by Xiao *et al*. (2017) (Supplementary Table 2). Fallopian tube fluid was collected from these same participants, and the concentration of *IL-6* and *TNFA* was detected with sandwich ELISA kits (Proteintech, USA.Cat.#KE00007 and Cat.#KE00068).

### Statistical analyses

Extraction of Gene Expression Exon microarrays is supported by Feature Extraction software (version 11.0.1.1; Agilent). Quantile normalization and data analyses were carried out using GeneSpring GX v12.1 software (Agilent Technologies). The threshold values for designating differentially expressed mRNAs were fold change >2 and *P* < 0.01. The microarray evaluation was executed by the Shanghai Biotechnology corporation, China (http://www.shbio.cn). The 2^-ΔΔCT^ method was used to analyze RT-qPCR data, and ELISA data were analyzed using GraphPad Prism 7 (GraphPad Software Inc.).

## Results

### Patient characteristics and experimental workflow

**Figure 1:**
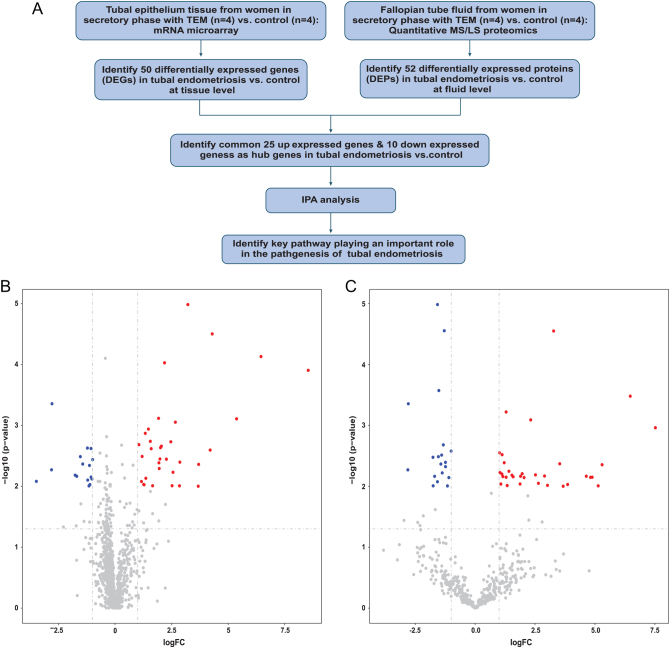
(A) Integrative genomic and proteomic analysis flow diagram. (B) Volcano plot displaying the differentially expressed genes (DEGs) cross-referenced with -log (*P*-value) (y-axis) and log_2_ FC (x-axis). (C) Volcano plot displaying the differentially expressed proteins (DEPs) cross referenced with -log (*P*-value) (y-axis) and log_2_ FC (x-axis). Fold change (FC) >2 and *P*-value <0.01 were identified as significantly altered.

The clinical characteristics of the recruited participants are shown in Table 1. No differences were found in age and gravidity between tubal EM patients and the control group. Figure 1A shows our workflow. After collecting tubal epithelium and fallopian tube fluid from women in the secretory phase of EM, we performed mRNA microarray analysis and quantitative MS/LS proteomics, respectively. We then identified DEGs and DEPs, and identified up-regulated and down-regulated genes before performing a bioinformatics analysis to determine key pathways that play an important role in the pathogenesis of endometriosis.

**Table 1:** Demographics and clinical pathological data for the experiment and validation samples.

Sample number	Age	Infertility	Tubal EM	Pathologic diagnosis	Recurrence in 1 year
N302837-TEM	43	√	√	Diffuse adenomyosis of the uterus and tubal endometriosis	×
N129922-TEM	47	√	√	Endometriotic cysts of the ovary and tubal endometriosis	×
N305896-TEM	45	×	√	Endometriotic cysts of the ovary and tubal endometriosis	×
N308632-TEM	42	×	√	Endometriotic cysts of the ovary and tubal endometriosis	×
N305044-NC	45	×	×	Uterine leiomyoma	×
N306224-NC	46	×	×	Uterine leiomyoma	×
N303534-NC	45	×	×	Uterine leiomyoma	×
N306515-NC	46	×	×	Uterine leiomyoma	×
E382856-TEM	43	×	√	Endometriotic cysts of the ovary and tubal endometriosis	×
E383026-TEM	47	×	√	Endometriotic cysts of the ovary and tubal endometriosis	×
E382986-TEM	38	×	√	Endometriotic cysts of the ovary and tubal endometriosis	×
E381882-TEM	38	√	√	Endometriotic cysts of the ovary and tubal endometriosis	×
E382981-NC	49	×	×	Uterine leiomyoma	×
E109737-NC	45	×	×	Uterine leiomyoma	×
E383093-NC	47	×	×	Uterine leiomyoma	×
E275821-NC	43	×	×	Uterine leiomyoma	×

E: Validation samples; N: Experiment samples; NC: Non-tubal endometriosis Control; TEM: Tubal Endometriosis.

### Differential mRNA profiles and functional analysis by IPA

**Figure 2:**
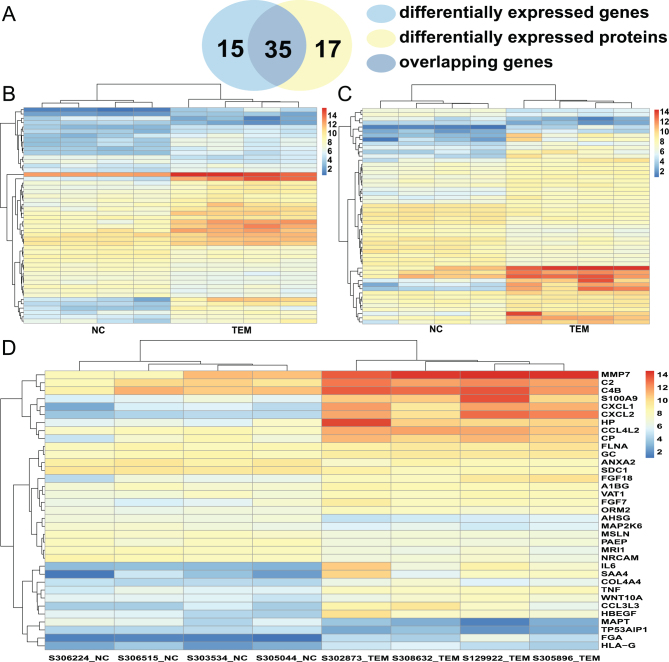
(A) Venn diagram displaying 35 commonly deregulated mRNAs/proteins from 50 DEGs and 52 DEPs. (B) Hierarchical heatmap of DEGs comparing patients with tubal endometriosis (tubal EM) and Non-endometriosis Control (NC). (C) Hierarchical heatmap of DEPs comparing patients with tubal EM and NC. (D) Hierarchical heatmap of 35 commonly deregulated mRNAs/proteins comparing patients with tubal EM and NC.

**Figure 3:**
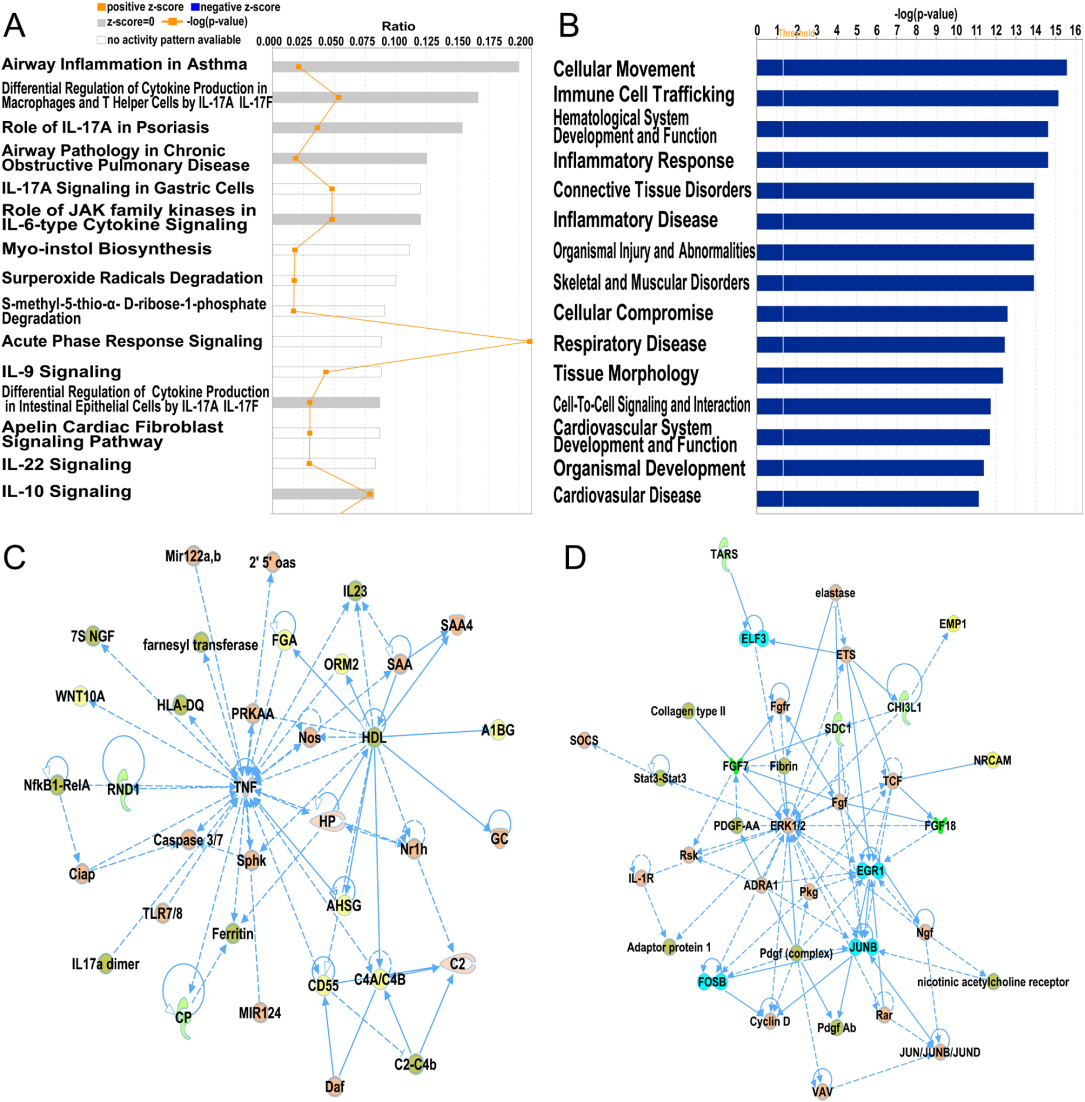
(A) Ingenuity Pathway Analysis (IPA) showing top 15 canonical pathways significantly modulated by DEGs among the tubal EM fallopian tube epithelium tissues compared to healthy subject tubal epithelium tissues (*P* < 0.05). The *P*-value for each pathway is indicated by the bar and is expressed as -log (*P*-value). The orange line represents the ratio of the number of genes in a given pathway that meet the cutoff criteria, divided by the total number of genes that comprise that pathway. (B) IPA showing top 15 diseases and biological functions of DEGs. The yellow threshold indicates 95% confidence. (C) The most significant molecular network by IPA analysis. Associated network functions are cellular compromise, inflammatory response, and neurological disease. (D) The second most significant molecular network. Associated network functions are cellular movement, neurological disease, and organismal injury and abnormalities.

To determine changes in gene expression, a microarray analysis was performed to investigate transcriptomes of fallopian tubal epithelia from both control individuals and EM patients. We detected a total of 22,787 genes, and after compared to LC-MS/MS detected proteins, we found commonly 1189 genes. Details for the annotation used for transcript identifiers were listed in Supplementary Table 3. Among them, 50 genes were found to be differentially expressed with a fold change >2 and *P* < 0.01 (shown in Supplementary Table 4), out of which 34 were up-regulated and 16 were down-regulated, as shown in the volcano plot (Fig. 1B) and heat map (Fig. 2B). Using IPA software, we examined the relationship between these significantly dysregulated genes to determine the most significant canonical pathways, diseases, and biological functions, as well as networks involved in tubal EM. The top 15 pathways enriched for 50 DEGs included Airway Inflammation in Asthma, Differential Regulation of Cytokine Production in Macrophages and T helper Cells by IL-17A and IL-17F, and Role of IL-17A in Psoriasis, and Airway Pathology in Chronic Obstructive Pulmonary Disease, as shown in Fig. 3A. The top 15 diseases and biological functions included Cellular Movement and Immune Cell Trafficking and Hematological System Development and Function, as shown in Fig. 3B. Also, IPA identified significant networks associated with the DEGs in tubal EM. These networks were scored based on the number of genes participating in any particular network. We identified five gene networks with scores from 15 to 27 genes. The top two of the five gene networks and their associated functions were cellular compromise, inflammatory response, and neurological disease (Fig. 3C) and cellular movement, neurological disease, and organismal injury and abnormalities (Fig. 3D).

### Differential protein profiles and functional analysis by IPA

**Figure 4:**
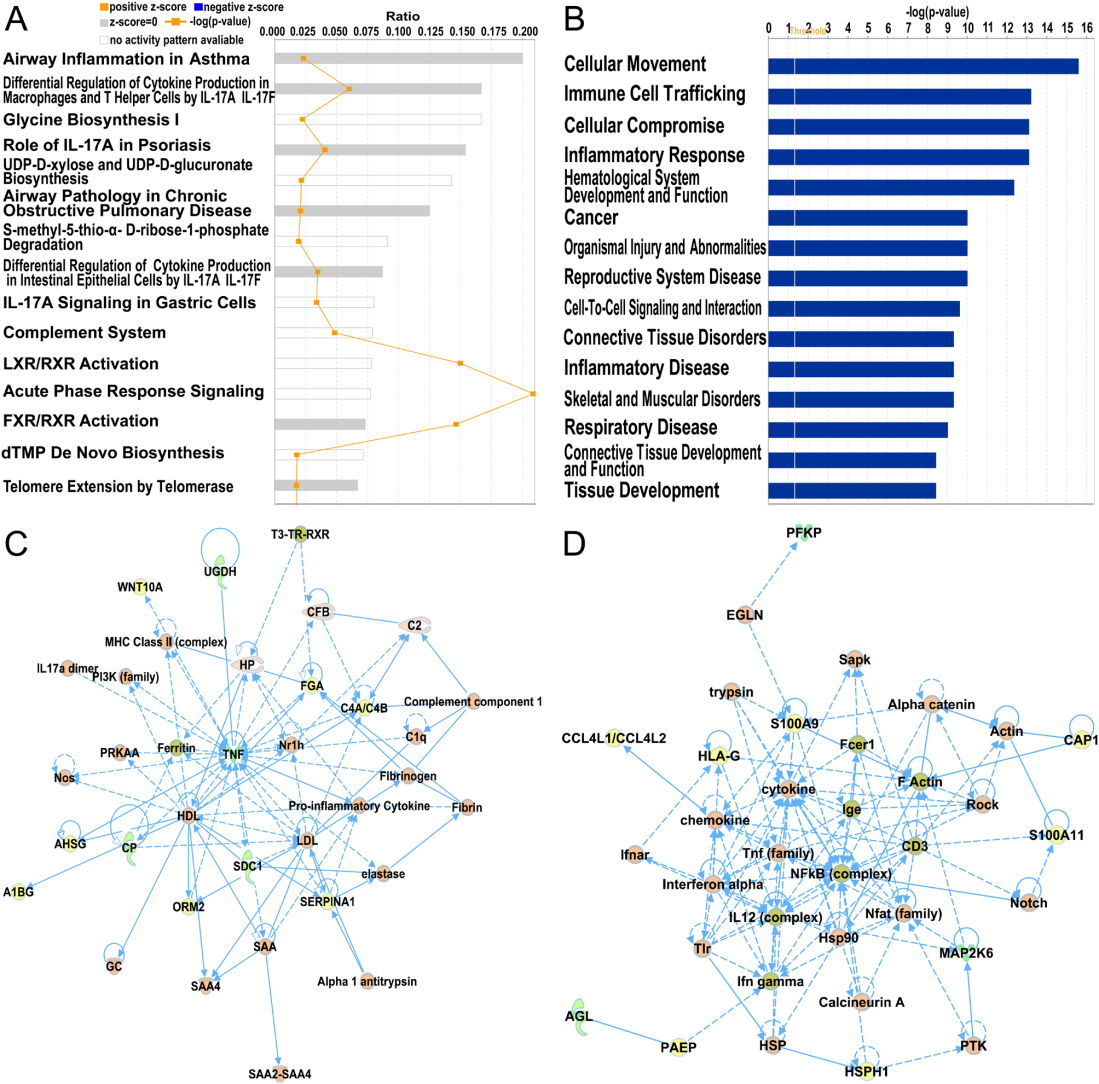
(A) IPA showing top 15 canonical pathways significantly modulated by DEPs among the TEM fallopian tube epithelium tissues compared to healthy subject tubal epithelium tissues (*P* < 0.05). The *P*-value for each pathway is indicated by the bar and is expressed as -log (*P* value). The orange line represents the ratio of the number of genes in a given pathway that meet the cutoff criteria divided by the total number of genes that comprise that pathway. (B) IPA showing top 15 diseases and biological functions of DEPs. The yellow threshold indicates 95% confidence. (C) The most significant molecular network by IPA analysis. Associated network functions are metabolic disease, organismal injury, and abnormalities and inflammatory disease. (D) The second most significant molecular network. Associated network functions are dermatological diseases and conditions, organismal injury and abnormalities, and cellular compromise.

A total of 1483 proteins were detected by quantitative label-free LC-MS/MS. Our proteomic analysis identified 52 DEPs in common between the control group and tubal EM group using unpaired *t*-test (shown in Supplementary Table 5). Details for the annotation used for protein identifiers were shown in Supplementary Table 6. Among these proteins, 33 were over-expressed and 19 were under-expressed in the fallopian tube fluid collected from tubal EM patients, as shown in Figs 1C and 2C. After identification of DEPs, we performed functional annotation using IPA software. Fig. 4A revealed the top 15 pathways enriched for 52 DEPs, such as Airway Inflammation in Asthma, Differential Regulation of Cytokine Production in Macrophages and T helper Cells by IL-17A and IL-17F, and Glycine Biosynthesis I. The top 15 diseases and biological functions included Cellular Movement and Immune Cell Trafficking and Cellular Compromise, as shown in Fig. 4B. Also, IPA identified significant networks associated with the DEPs in tubal EM. We identified five gene networks with scores from 6 to 39 genes. In Fig. 4, we present the top two of the five protein networks and their associated functions: metabolic disease, organismal injury and abnormalities, and inflammatory disease (Fig. 4C); and dermatological diseases and conditions, organismal injury and abnormalities and cellular compromise (Fig. 4D).

### Transcriptome-proteome integration and functional analysis by IPA

**Figure 5:**
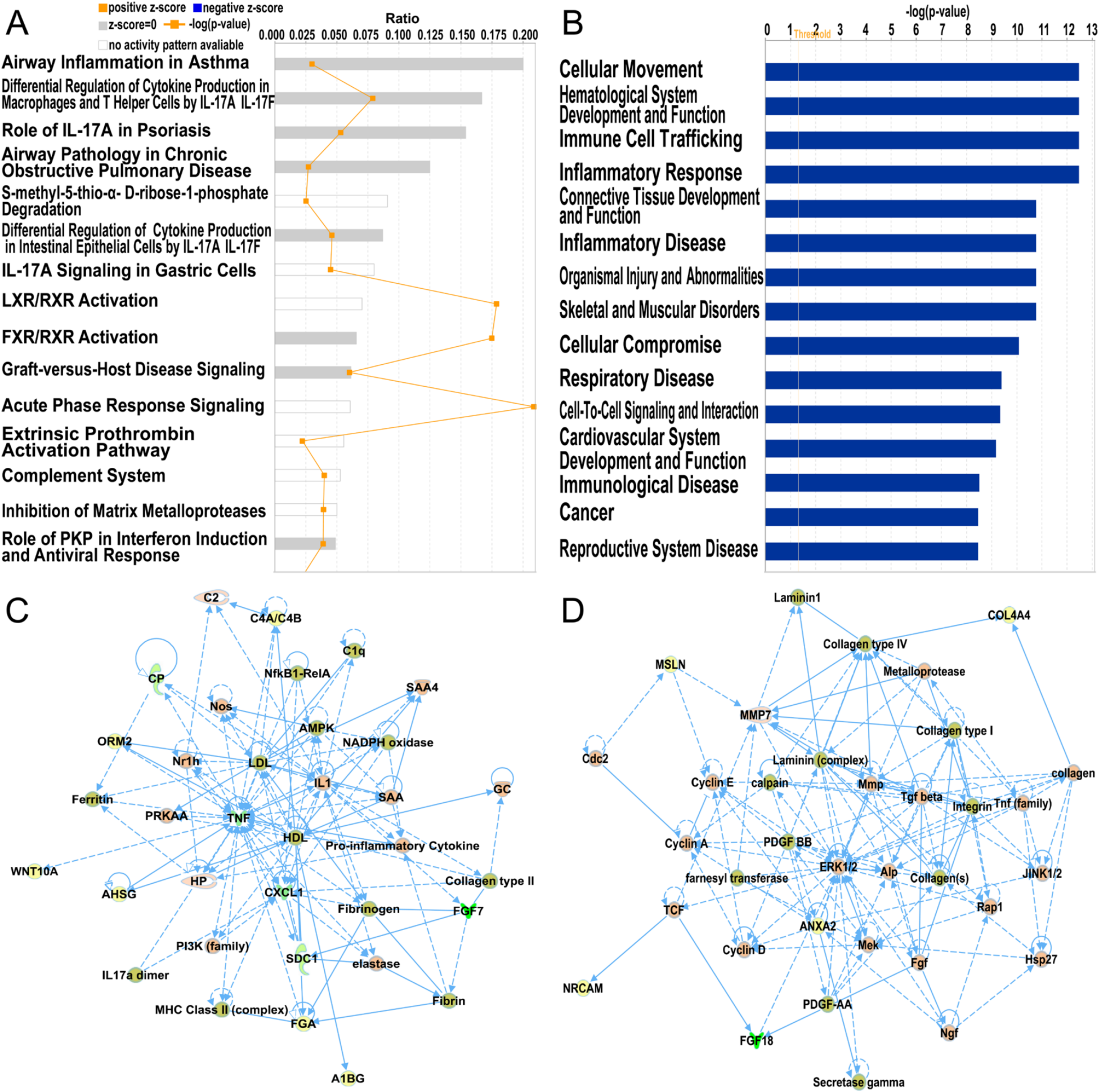
(A) IPA showing top 15 canonical pathways significantly modulated by 35 commonly deregulated mRNAs/proteins among the TEM fallopian tube epithelium tissues compared to healthy subject tubal epithelium tissues (*P* < 0.05). The *P*-value for each pathway is indicated by the bar and is expressed as -log (*P* value). The orange line represents the ratio of the number of genes in a given pathway that meet the cutoff criteria divided by the total number of genes that comprise that pathway. (B) IPA showing top 15 diseases and biological functions of commonly deregulated mRNAs/proteins. The yellow threshold indicates 95% confidence. (C) The most significant molecular network by IPA analysis. Associated network functions are inflammatory disease, connective tissue, and disorders and immunological disease. (D) The second most significant molecular network. Associated network functions are cancer, organismal injury and abnormalities, and gastrointestinal disease.

We compared mRNAs and proteins found to be dysregulated in both tubal EM samples and their corresponding normal controls in order to identify overlapping features of transcriptomes and proteomes. Among the 50 DEGs and 52 DEPs, we observed 35 commonly dysregulated mRNAs/proteins, including 25 up-regulated and 10 down-regulated genes (Fig. 2A and D). We then conducted an analysis of canonical pathways, biological functions, and interaction networks on these 35 overlapping mRNAs/proteins using IPA software. The top 15 pathways based on ratio (-log(*P*-value)) included Airway Inflammation in Asthma, Differential Regulation of Cytokine Production in Macrophages and T helper Cells by IL-17A and IL-17F, and Role of IL-17A in Psoriasis, as shown in Fig. 5A. The top 15 diseases and biological functions included Cellular Movement, Hematological System Development and Function, and Immune Cell Trafficking, as shown in Fig. 5B. Significant networks associated with the 35 overlapping dysregulated mRNAs/proteins in tubal EM were determined via IPA. These networks were scored based on the number of genes participating in any particular network. We identified five gene networks with scores from 5 to 36 genes. In Fig. 5, we present the top two of the five gene networks and their associated functions: inflammatory disease, connective tissue disorders, and immunological disease (Fig. 5C) and cancer, organismal injury, and abnormalities and gastrointestinal disease (Fig. 5D).

**Figure 6:**
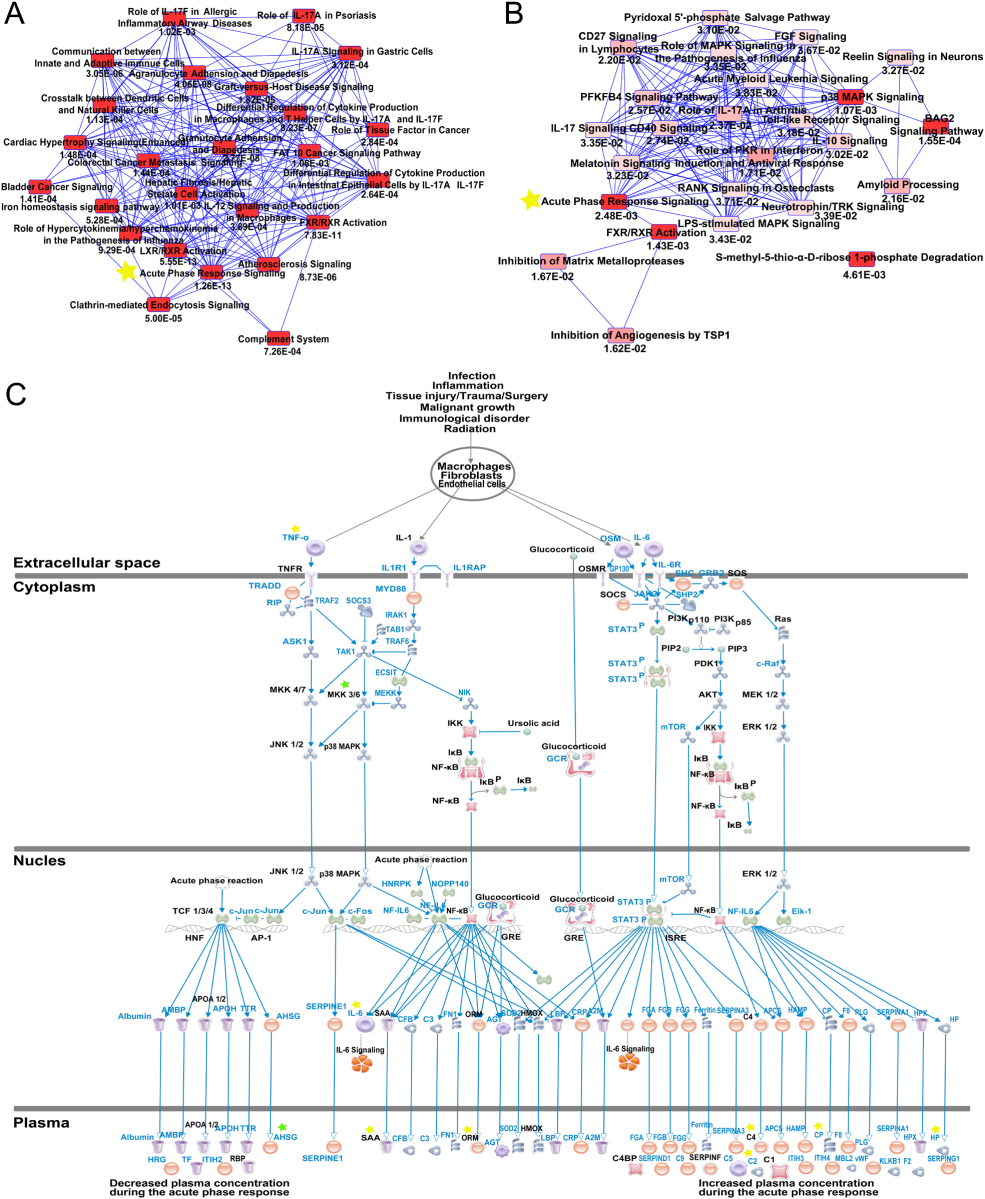
Pathway analysis of commonly dysregulated genes. (A) Overlapping canonical pathways map with annotated *P*-values representing shared biology among the 25 commonly up-regulated mRNAs/proteins, as determined through IPA. Connected canonical pathways share one or more genes in common. The brighter the red of the node, the more significant the canonical pathway in the gene set. The yellow star highlights the overlapping pathway between 25 commonly up-regulated gene set enriched pathways and 10 commonly down-regulated gene set enriched pathways. (B) Overlapping canonical pathways map with annotated *P*-values representing shared biology among the 10 commonly down-regulated mRNAs/proteins by IPA. (C) Details of Acute Phase Response Signaling pathways. Eight commonly up-regulated mRNAs were highlighted with yellow stars and two down-regulated mRNAs with green stars.

Canonical pathways enriched for 25 commonly up-regulated and 10 commonly down-regulated mRNAs/proteins were also determined using the IPA software. Overlapping canonical pathway maps represent shared biology with *P*-values annotated for the 25 commonly up-regulated genes (Fig. 6A) and 10 commonly down-regulated genes (Fig. 6B). Connected canonical pathways share one or more genes in common. Acute phase response signaling and FXR/RXR activation are common canonical pathways for overlapping up-regulated and down-regulated gene sets. We selected the Acute phase response signaling pathway as the most relevant pathway for tubal EM, since it ranks the top canonical pathway according to *P*-value. A total of eight up-regulated (*IL6*, *C2*, *C4B*, *CP*, *HP*, *SAA4*, *TNFA* and *ORM2*) and two down-regulated genes (*AHSG* and *MAP2K6*) from this dataset were mapped to the acute phase response signaling pathway, which is shown in Fig. 6C.

### Validation of mRNAs and proteins critical to tubal EM

**Figure 7:**
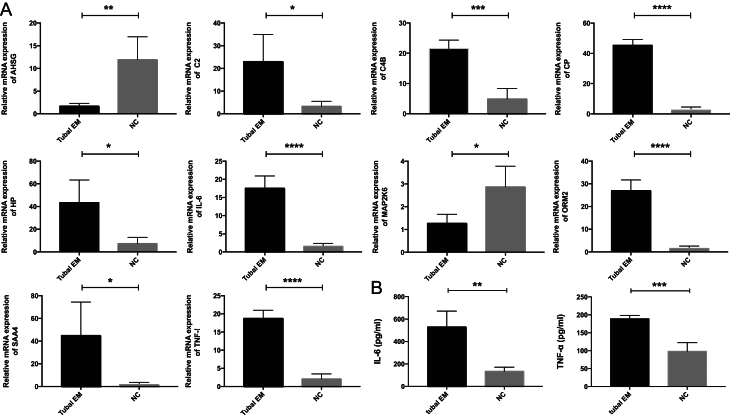
(A) Verification of the expression of eight commonly up-regulated and ten commonly down-regulated genes in fallopian tube epithelium tissues from four patients with tubal EM and four participants without tubal EM by quantitative real-time polymerase chain reaction (qRT-PCR). (B) The protein expression of *IL-6* and *TNFA* in fallopian tube fluid with ELISA. Results were expressed as mean ± s.d., and statistical significance was showed as *, *P* < 0.05; **, *P* < 0.01; ***, 0.0001 < *P* < 0.01; ****, *P* < 0.0001.

Real-time PCR was conducted to validate the transcription of ten genes selected from microarray analysis, including eight commonly up-regulated genes and two commonly down-regulated genes. Consistent with the microarray analysis results, compared with the non-tubal EM group, the tubal EM group exhibited significantly increased expression of *CP*, *IL6*, *ORM2*, and *TNFA* (*P* < 0.0001), *C2*, *C4B*, *HP*, and* SAA4* (*P* < 0.05) and decreased expression of *AHSG* and *MAP2K6* (*P* < 0.05) in fallopian tube epithelium using *GAPDH* as reference gene (Fig. 7A). Compared with the non-tubal EM group, the tubal EM group exhibited significantly increased expression of *IL6*, *ORM2*, and *TNFA* (*P* < 0.0001), *C2*, *C4B*, *CP*, *HP*, and* SAA4* (*P* < 0.01) and decreased expression of *AHSG* and *MAP2K6* (*P* < 0.01) in fallopian tube epithelium using *ACTB* as reference gene (Supplementary Fig. 1A). Compared with the non-tubal EM group, the tubal EM group exhibited significantly increased expression of* C2*, *C4B*, *CP*, *HP*, *IL6*, *ORM2*, *SAA4*, and *TNFA* (*P* < 0.05) and decreased expression of *AHSG* (*P* < 0.05) and *MAP2K6* (*P* < 0.01) in fallopian tube epithelium using *18s RNA* as reference gene (Supplementary Fig. 1B). ELISA was performed to validate the expression of two up-regulated proteins selected from quantitative label-free LC-MS/MS analysis. ELISA results showed that the protein level of *IL6* (*P* < 0.01) and *TNFA* (*P* < 0.001) had the same expression pattern as their label-free LC-MS/MS analysis in fallopian tube fluid (Fig. 7B).

## Discussion

The fallopian tube is the part of the female reproductive tract that hosts fertilization and pre-implantation development of the embryo, and tubal epithelial cells play a dominate role in the production of tubal fluid. Changes in tubal fluid concentration and content can alter fluid viscosity, hence, influencing the flow rate of the fluid, which can be sensed by tubal epithelial cells (Andrade *et al*. 2005). Effective crosstalk between the fallopian tube epithelium and tubal fluid is crucial for optimal functioning of the fallopian tube. There are several subtypes of EM, classified according to the location of ectopic endometrial glands and stroma, and tubal EM is one of these. Over the past few decades, researchers have focused on understanding the pathogenesis of EM and have proposed many models. EM is thought to result from complex genetic, immunological, hormonal, and environmental factor interactions (Koninckx *et al*. 2019). Combined transcriptomics and proteomics analysis can provide insight into the mechanisms of diseases in a more comprehensive way. Researchers found DEGs in menstrual endometrium compared with luteal endometrium in EM patients and five peptide peaks enabling diagnosis of EM by integrated mRNA microarray and proteomic of eutopic endometrium of women with and without EM (Fassbender *et al*. 2012). A study found effects of selenium status on gene expression and proteomics profiles of rectal biopsies combining transcriptomic and proteomic approaches (Meplan *et al*. 2016). Another study integrated transcriptomics and proteomics to explore DEGs between the E7.5 and E9.5 placenta and DEPs unique to the protein profiling. Then they found novel genes predicted to be related to placental metabolism (Abdulghani *et al*. 2019).

However, few studies have looked specifically at tubal EM. In this study, we combined microarray, proteomic, and bioinformatics approaches to provide new insights into tubal EM. We first profiled mRNA expression in fallopian tube epithelium and protein expression in tubal fluid to identify common differentially expressed mRNAs/proteins. We found that these mRNAs/proteins were mainly involved in inflammatory interactions, showing a similar functional pattern to that seen for EM. Compared with other subtypes, the common dysregulated mRNAs/proteins in tubal EM were mainly enriched for acute phase response signaling, FXR/RXR activation, and inflammation-related pathways. This result agrees with the fact that EM is often characterized as a disease of inflammation, due to ectopic endometrial tissue activation of the immune response. In addition, the estrogen proliferation that these ectopic lesions are noted to cause can further promote inflammation (Zhao *et al*. 2015). Moreover, even with estrogen suppression, EM patients still experience increased levels of inflammation accompanied by symptoms (Zhao *et al*. 2015). Inflammation, which changes the microenvironment of the fallopian tube, is thought to contribute to the pathogenesis of tubal EM and may increase disease severity. It is well known that fertility decreases significantly with female age. For tubal EM patients, the older they are, the higher levels of inflammatory cytokines and complement components may exist in pelvic microenvironment. It suggests that women with tubal EM may have potential of successful pregnancy if diagnosed at early age and asked for assisted reproductive technology to lower its harmful effect on reproduction.

Consistent with former studies, some commonly up-regulated mRNAs/proteins that we identified in our comparative study were EM-associated. Previous studies have found that E_2_/ERα/IL-6 cross-talk plays a crucial role during early initiation of EM, while *IL-6* influences the number of lesions formed and E_2_ plays a role in lesion growth (Burns *et al*. 2018). Another study found that hypoxia-induced elevated levels of *IL-6* in ectopic lesions caused aberrant activation of STAT3 signaling pathway and contributed to EM cell survival under ectopic environmental conditions (Hsiao *et al*. 2017). In addition, *IL-6* levels in the peritoneal fluid were higher in patients with EM than in the control group, and EM severity was proportionate to *IL-6* expression level (De Andrade *et al*. 2017). Endometriotic lesions exist in an inflammatory microenvironment with higher local concentrations of cytokines, such as tumor necrosis factor α (*TNFA*) (Kocbek *et al*. 2016). *TNFA* increases expression of phosphorylated *IKKB*, an important protein present in multiple signaling pathways that influences gene transcription, proliferation, and apoptosis (Kocbek *et al*. 2016). Cytokine assays showed that *TNFA* concentrations were higher in embryos incubated with EM-peritoneal fluid than in those incubated with non-EM peritoneal fluid, which significantly decreased the rate of blastocyst development and increased apoptotic cell percentage in embryos. Furthermore, treatment of embryos with *TNFA* retarded development and induced apoptosis (Chen *et al*. 2013). Another study showed that *C2* and *C4B*, components of complement pathways, had significantly higher expression levels in ectopic tissues compared with control endometrium (Ahn *et al*. 2016). Researchers also have integrated mRNA microarray and proteomic of eutopic endometrium of women with EM and without EM, finding that *C2* and *TNFA* were differentially expressed, which were in line with our data (Fassbender *et al*. 2012). In addition, a commonly down-regulated mRNAs/protein was also reportedly involved in the pathogenesis of EM. One study indicated that the *AHSG* polymorphism was associated with EM in Korean women (Kim *et al*. 2004). The acute phase response is a rapid inflammatory response that provides protection against microorganisms through non-specific defense mechanisms. In addition to infection, the acute phase response can also be triggered by tissue injury, neoplastic growth, and immunological disorders. Typically, it consists of an increase of inflammatory factors (such as pro-inflammatory cytokines) and a concentration change of several plasma proteins (the acute phase proteins) largely due to an altered hepatic metabolism. The up-regulation of *MMP7* has previously been shown to promote the epithelial-mesenchymal transition during ovarian EM progression (Chatterjee *et al*. 2018), which agrees with our results. Positive acute phase response proteins play roles in opsonization and trapping of micro-organisms, in addition to complement activation, neutralizing enzymes, and modulating the immune response. In EM patients, the concentrations of acute phase proteins, complement factors, and immunoglobulins in the abdominal cavity are likely to be increased secondary to changes in vascular permeability (Dunselman *et al*. 1988). In accordance with these previous studies, we found that *IL-6*, *TNFA*, *C2*, *C4B*, *MMP7*, and* AHSG* were preferentially expressed both in tubal epithelium tissues and tubal fluid from patients with tubal EM, as compared with healthy control, and that this acute phase response may play a role in the pathogenesis of tubal EM.

We also identified novel mRNAs/proteins, such as *ORM2*, *SAA4*, *CP*, *HP* and* MAP2K*6, which have not previously been associated with EM. *ORM2* was founded to be associated with microglial interaction (Jo *et al*. 2017),* SAA4* was identified as a rheumatoid arthritis-screening biomarker (Seok *et al*. 2017), and *MAP2K6* was reported to be involved in the pathogenesis of colorectal adenocarcinoma (Rasmussen *et al*. 2016). The differences between mRNA expression in tubal epithelium and protein expression in fallopian tube fluid could result from post-translational modification and secretion. Interestingly, when comparing our data with ovarian EM (Vehmas *et al*. 2014), we found *AHSG* was the only overlapping gene, and when compared with deep EM (Matsuzaki *et al*. 2004), we found there are no overlapping genes. Two explanations for this discrepancy are possible. Primarily, normal tubal epithelium from paired patients was used as control in our study, while others chose matched eutopic endometrium from the same patients as control. In addition, we focus on revealing the common genes/proteins between tubal epithelium and tubal fluid in terms of integrated transcriptome and proteome, while others’ findings were based on a single dimension of analysis. All these DEGs (*IL6*, *TNFA*, *C4B*, *C2*, *CP*, *HP*, *SAA4*, *ORM2*, *AHSG* and *MAP2K6 et al*.) may contribute to the pathogenesis of tubal EM through the acute phase response signaling pathway. Thus, in the future study, it is of great importance to explore the exact roles, such as the interactions between these DEGs and ciliary beat frequency of fallopian tube epithelium, how these DEGs affect the differentiation rate of fallopian tube ciliary epithelium, and how these DEGs change the muscular contractions. And, DEPs that correspond to these DEGs may serve as biomarkers for the detection of tubal EM. As for the DEPs that do not correspond to these DEGs, we can explore the post-translational modifications such as phosphorylation and carbonylation.

Importantly, the abovementioned studies utilized different research methods and varying amounts of patient samples, along with different conclusions. Our investigation, utilizing microarray, proteomics, and bioinformatics methods, will contribute to a better understanding of the molecular pathology of tubal EM and may provide useful strategies to diagnose and treat complex cases, with the goal of reducing morbidity and disease complications such as infertility. However, a limitation of our study is the small sample size. Although FDR-adjusted *P*-value of <0.01 was utilized as the cutoff criterion to exclude false-positives, it still calls for further recruiting tubal EM patients and control group.

## Supplementary Material

Table S1. The RINs for the samples and the concentration of RNA used for the hybridization.

Table S2. Primer sequence information for RT-qPCR used in this study.

Table S3. Details for the annotation used for transcript identifiers.

Table S4. 50 Differentially expressed genes microarray information

Table S5. 52 Differentially expressed proteins LC-MS/MS information

Table S6. Details for the annotation used for protein identifiers.

Fig S1

## Declaration of interest

The authors declare that there is no conflict of interest that could be perceived as prejudicing the impartiality of the research reported.

## Funding

This research did not receive any specific grant from any funding agency in the public, commercial, or not-for-profit sector.

## Author contribution statement

Hang Qi performed experiments, analyzed the data, and wrote the paper. Huiyu Zhang, Xiaoya Zhao, Ya Qin, Guiling Liang, and Xiaoqing He performed investigation. Jian Zhang conceived the study and reviewed and edited the paper.
