# Supplementary material for: Integrated analysis of mRNA and protein expression profiling in tubal endometriosis
Source: Reproduction. 2020 Mar 2;159(5):601–14. doi: 10.1530/REP-19-0587 (PMC7159149; doi:10.1530/REP-19-0587)
Supplement: Table S1. The RINs for the samples and the concentration of RNA used for the hybridization. [file supplementary_table_1.pdf]

Table S1. The RINs for the samples and the concentration of RNA used for the hybridization.

**Sample name**	**Concentration (ng/ul)**	**Volume (ul)**	**Total (ug)**	**A260/A280**	**2100 Result**	
					**RIN***	**28S/18S**
N302837-TEM	590.8	55	32.49	2.13	7.0	1.9
N129922-TEM	583.4	70	40.84	2.03	7.6	1.0
N305896-TEM	569.1	55	31.30	2.16	8.5	1.7
N308632-TEM	651.5	55	35.83	2.17	8.8	1.4
N305044-NC	506.3	55	27.85	2.11	8.2	1.9
N306224-NC	542.3	55	29.83	2.09	8.5	1.7
N303534-NC	533.2	55	29.33	2.15	8.6	1.7
N306515-NC	574.1	55	31.58	2.13	9.3	1.8

*: RNA Integrity Number. From 0-10, the higher the score, the better the RNA integrity.
