# Supplementary material for: Integrated analysis of mRNA and protein expression profiling in tubal endometriosis
Source: Reproduction. 2020 Mar 2;159(5):601–14. doi: 10.1530/REP-19-0587 (PMC7159149; doi:10.1530/REP-19-0587)
Supplement: Table S2. Primer sequence information for RT-qPCR used in this study. [file supplementary_table_2.pdf]

Table S2. Primer sequence information for RT-qPCR used in this study.

Symbol	Gene name	Accession number	Forward and reverse primer sequence [5′-3′]	Production size
GAPDH	Glyceraldehyde	NM_002046.5	F: TCCAAAATCAAGTGGGGCGA<br>R: TGATGACCCTTTTGGCTCCC	115 bp
ACTB	β-actin	NM_001101.3	F: CTTCCAGCCTTCCTTCCTGG<br>R: CTGTGTTGGCGTACAGGTCT	110 bp
18s RNA	18s RNA	M10098.1	F: GGAGCCTGCGGCTTAATTTG<br>R: CCACCCACGGAATCGAGAAA	100 bp
AHSG	alpha 2-HS glycoprotein	NM_001354571.2	F: CCCAGGGCTGATTTATAGACAA<br>R: GTTCAAGGTGTGTTTGTATCCC	118bp
C2	Complement C2	NM_000063.6	F: CTCCCTCCTGCCCTCAGAACG<br>R: GCCACTGTCCGCTGCTCTTG	150bp
C4B	Complement C4B	NM_001002029.4	F: ACACTGCCTCCCGAGACCAAG<br>R: CGTGACAACCAAGCCGCATAGG	110bp
CP	Ceruloplasmin	NM_000096.4	F: GGGCCAATGAAAATATGCAAGA<br>R: CTGATCAGGTGCAGTTGTAAAC	156bp
HP	Haptoglobin	NM_005143.5	F: AGGCATTATGAAGGCAGCACAGTC<br>R: CGCATCGCCATAGCAGGTGTC	135bp
IL-6	Interleukin 6	NM_000600.5	F: CACTGGTCTTTTGGAGTTTGAG<br>R: GGACTTTTGTACTCATCTGCAC	101bp
MAP2K6	Mitogen-activated protein kinase kinase 6	NM_002758.4	F: CAAGGCTTGCATTTCTATTGGA<br>R: CAAATCCATCAGTAGCCGTTTC	196bp
ORM2	Orosomucoid 2	NM_000608.4	F: GACACGATCTTTCTCAGAGAGT<br>R: CTTGTCAGCATAGAAAGACAGC	124bp
SAA4	Serum amyloid A4	NM_006512.4	F: GGGTGTCTGGGCTGCTAAACTC<br>R: GCCCCATTCCTCAGCTTTCTCG	133bp
TNFA	Tumor necrosis factor	NM_000594.4	F: CGTGGAGCTGGCCGAGGAG<br>R: GCAGGCAGAAGAGCGTGGTG	125bp
