# Supplementary material for: Integrated analysis of mRNA and protein expression profiling in tubal endometriosis
Source: Reproduction. 2020 Mar 2;159(5):601–14. doi: 10.1530/REP-19-0587 (PMC7159149; doi:10.1530/REP-19-0587)
Supplement: Table S3. Details for the annotation used for transcript identifiers. [file supplementary_table_3.pdf]

Table S3. Details for the annotation used for transcript identifiers.

Probe Name	Gene Symbol	Ensembl	Accession	Probe Seq
A_23_P38816	*A1BG*	ENST00000598345	NM_130786	CCTGGTGGCAGAAAGCTGATGCAGCCGCGGG CCCAGAGGTGCTGTTGGTGTCCTCAGAAG
A_23_P117599	*AHSA1*	ENST00000556866	NM_012111	ATTAGTGTGAGCCTTGCCAAAGATGAGCCTG ACACAAATCTCGTGGCCTTAATGAAGGAA
A_23_P155509	*AHSG*	ENST00000411641	NM_001622	CATGATTGTGTTCTCTGCCTCTGGTTGACCTT ACAAAAACCATTGGAACTGTGACTTTGA
A_23_P205959	*ALDH1A3*	ENST00000329841	NM_000693	ATATAGACACTAACCTTGATAGTGATACGTT AGAGGGTTCCTATTCTTCCATTGTACGAT
A_32_P148345	*ANXA2*	ENST00000421017	NM_001002857	TAAGCCAAAGAAATGTACATTCCAAGCAGTT GGAAGTGAAATCTATGATGTGAAACACTT
A_23_P48217	*APOLD1*	ENST00000326765	NM_030817	GAGTGGTTGTAGAAGTCTCCCTAAATCAGAC ATGTCAAGCAATCAGCCAACGTGGTGTAT
A_32_P162183	*C2*	ENST00000299367	NM_000063	GGTTGACTTGACTCATGCTTGTTTCACTTTCA CATGGAATTTCCCAGTTATGAAATTAAT
A_23_P42282	*C4B*	ENST00000463249	NM_001002029	GCTTTCCGCCTCTTTGAGACCAAGATCACCCA AGTCCTGCACTTCACCAAGGATGTCAAG
A_24_P228130	*CCL3L3*	ENST00000621940	NM_001001437	TTCCACAGAATTTCATAGCTGACTACTTTGAG ACGAGCAGCCAGTGCTCCAAGCCCAGTG
A_23_P207564	*CCL4L2*	ENST00000613947	NM_001291470	CAGGAAGTCTTCAGGGAAGGTCACCTGAGCC CGGATGCTTCTCCATGAGACACATCTCCT
A_23_P138760	*CLCF1*	ENST00000312438	NM_013246	TGGCAATTCTACACAAAAAGAGATGAGATTA ACAGTGCAGGGTTGGGGTCTGCATTGGAG
A_23_P254522	*COL4A4*	ENST00000396625	NM_000092	TCCTAAAAGTTCAACCTGTTCATCTTGAACTT GGCCTGAGAACATTTTCTGGGAAGAGGT
A_23_P26021	*COPS2*	ENST00000299259	NM_004236	TGCTTTTTTGATCAACTGGTTTGTGTTTTGCTG CTGCATTTATCCCAAGAAAAACAGCTT
A_23_P414793	*CP*	ENST00000463556	NM_000096	TCACGGCCATAGCTTCCAATACAAGCACAGG GGAGTTTATAGTTCTGATGTCTTTGACAT
A_23_P7144	*CXCL1*	ENST00000395761	NM_001511	CATACTGCCTTGTTTAATGGTAGTTTTACAGT GTTTCTGGCTTAGAACAAAGGGGCTTAA
A_23_P315364	*CXCL2*	ENST00000508487	NM_002089	CAATGTGACGGCAGGGAAATGTATGTGTGTC TATTTTGTAACTGTAAAGATGAATGTCAG
A_23_P108415	*DARS*	ENST00000537273	NM_001349	TTGATTTGGAGAAAATTAAGGCTTACATTGAT TCCTTCCGCTTTGGAGCCCCTCCTCATG
A_23_P12405	*ESPN*	ENST00000377828	NM_031475	GCCTCATTTCTTATCCCCGCCAAGGGTTTCCT CTCAGTCATTTGTTTACCAGAAACATGA
A_23_P375372	*FGA*	ENST00000403106	NM_021871	GGAGAGAGTACCACACAGAAAAACTGGTCAC TTCTAAAGGAGATAAAGAGCTCAGGACTG
A_23_P93027	*FGF18*	ENST00000274625	NM_003862	GGGTGATAAGGATTTTATTGTTGACTTGAAAC CCCCGATGACAAAAGACTCACGCAAAGG
A_24_P99244	*FGF7*	ENST00000560765	NM_002009	TCTACAGATCATGCTTTCACATTATCTGTCTA GTGGGTACTATATCTTTAGCTTGCAATG
A_23_P96568	*FLNA*	ENST00000498491	NM_001456	TCCCTCGGGAGAATGGCGTTTACCTGATTGAC GTCAAGTTCAACGGTACCCACATCCCTG
A_23_P167349	*GC*	ENST00000503472	NM_000583	CTTTACTGTGATTCAGAGATTGATGCTGAATT GAAGAATATCCTGTAGTCCTGAAGCATG
A_24_P140608	*HBEGF*	ENST00000230990	NM_001945	CCCATCTGTAGTAATTTATTGTCTGTCTACAT

				TTCTGCAGATCTTCCGTGGTCAGAGTGC
A_24_P311926	*HLA-G*	ENST00000428701	NM_002127	TTCCTGTTCCAGAAAAGGGGCTGGGATGTCT CCGTCTCTGTCTCAAATTTGTGGTCCACT
A_23_P206760	*HP*	ENST00000566821	NM_005143	GATAAGATGTGGTTTGAAGCTGATGGGTGCC AGCCCTGCATTGCTGAGTCAATCAATAAA
A_23_P71037	*IL6*	ENST00000485300	NM_000600	ATTGTTGTTGTTAATGGGCATTCCTTCTTCTG GTCAGAAACCTGTCCACTGGGCACAGAA
A_23_P5131	*ISYNA1*	ENST00000577820	NM_016368	TACCCTATGTTGAACAAGAAAGGACCGGTAC CCGCTGCCACCAATGGCTGCACCGGTGAT
A_24_P416489	*MAP2K6*	ENST00000590474	U39657	TGGGTAAATATACCCACAGGTTCTATGATTTG TAGCTCTAGGTTTCTTGATGATCAAGGA
A_23_P207699	*MAPT*	ENST00000621329	NM_016835	ACCAGTTCTCTTTGTAAGGACTTGTGCCTCTT GGGAGACGTCCACCCGTTTCCAAGCCTG
A_23_P52761	*MMP7*	ENST00000260227	NM_002423	TTGGGTATGGGACATTCCTCTGATCCTAATGC AGTGATGTATCCAACCTATGGAAATGGA
A_24_P279797	*MRI1*	ENST00000319545	NM_001031727	TTTTATGTGTAAAGATCATAAAACGTTGTAAG TTTTCTAAAACACATAAGCTCTCAATAA
A_23_P77529	*MSLN*	ENST00000545450	NM_005823	GTGGAATGTGACGTCCCTGGAGACCCTGAAG GCTTTGCTTGAAGTCGACAAAGGGCACGA
A_24_P408772	*NAMPT*	ENST00000354289	NM_005746	ACAGGCACCACTAATAATCAGACCTGATTCT GGAAACCCTCTTGACACTGTGTTAAAGGT
A_23_P127584	*NNMT*	ENST00000299964	NM_006169	CTGCTGTGAAAGAGGCTGGCTACACAATCGA ATGGTTTGAGGTGATCTCGCAAAGTTATT
A_24_P252364	*NRCAM*	ENST00000351718	NM_001037132	GTGGTTTTAAAAGTTCCTCTCTCTTTTGTAATT TATGTTCCCAATTGAGTGTGAATGTCC
A_23_P9485	*ORM2*	ENST00000431067	NM_000608	GTTTGGTTCCTACCTGGACGATGAGAAGAAC TGGGGGCTGTCTTTCTATGCTGACAAGCC
A_23_P257129	*PAEP*	ENST00000371768	NM_002571	TACGGTGGCGAACGAGGCCACGCTGCTCGAT ACTGACTACGACAATTTCCTGTTTCTCTG
A_23_P74001	*S100A12*	ENST00000368737	NM_005621	TGAAGGCTTTTTACCCAGCAATGTCCTCAATG AGGGTCTTTTCTTTCCCTCACCAAAACC
A_23_P23048	*S100A9*	ENST00000368738	NM_002965	GAGCTGGTGCGAAAAGATCTGCAAAATTTTC TCAAGAAGGAGAATAAGAATGAAAAGGTC
A_23_P87238	*SAA4*	ENST00000615694	NM_006512	GATATCCAATCACCAAAATTCAAACAGATAT CTCTATGCTCGGGGAAACTATGATGCTGC
A_23_P16944	*SDC1*	ENST00000381150	NM_001006946	ACCCTGGGCCCTGGGCTGGAATCAGGAATAT TTTCCAAAGAGTGATAGTCTTTTGCTTTT
A_23_P134176	*SOD2*	ENST00000541573	NM_001024465	TTGATGTGTGGGAGCACGCTTACTACCTTCAG TATAAAAATGTCAGGCCTGATTATCTAA
A_23_P107206	*STAT3*	ENST00000404395	NM_213662	TCATCATGGGCTATAAGATCATGGATGCTAC CAATATCCTGGTGTCTCCACTGGTCTATC
A_23_P70168	*TARS*	ENST00000502553	NM_152295	CTGCAGGCGTAACTATTTTTGACCTAGTCAGT TTTTAAACAATGTGCATTTGAAGGAGTT
A_24_P142118	*THBS1*	ENST00000260356	NM_003246	AATAAGCTGCTCTGCCCCTTGTGCTCAGAGTG GATGTTATGGGATTCCTTTTTTCTCTGT
A_23_P376488	*TNF*	ENST00000376122	NM_000594	GGGGTATCCTGGGGGACCCAATGTAGGAGCT GCCTTGGCTCAGACATGTTTTCCGTGAAA
A_24_P152398	*TP53AIP1*	ENST00000621818	NM_001195195	ACACACACACCTGGCTGGGTTTCAGATCCCTT AGTTTTGGGTGCCCAAGTTCACGGAGGG
A_23_P26954	*VAT1*	ENST00000592388	NM_006373	TGCCCCAACCATTGCAATGGGAGGTGGAGGG

				ACAGGCTCAGCCTCCTCATTGTCTAAATG
A_23_P102113	*WNT10A*	ENST00000258411	NM_025216	AACAAATATTTATTTTGCACTCTCTTTGCGGC ACTCTGGGGGCGGTGGGGTGCGTGGGGG
