# Supplementary material for: Integrated analysis of mRNA and protein expression profiling in tubal endometriosis
Source: Reproduction. 2020 Mar 2;159(5):601–14. doi: 10.1530/REP-19-0587 (PMC7159149; doi:10.1530/REP-19-0587)
Supplement: Table S4. 50 Differentially expressed genes microarray information [file supplementary_table_4.pdf]

Table S4. 50 Differentially expressed genes microarray information

Gene	FDR-adjusted p-value	Fold Change relative to control	S129922_TEM	S302873_TEM	S305896_TEM	S308632_TEM	S303534_NC	S305044_NC	S306224_NC	S306515_NC
A1BG	0.00944872	2.41096872	9.08865384	10.0553601	9.10764472	8.99075892	8.10122992	8.3054691	7.8861874	7.75934741
AHSA1	0.0024016	0.47443258	7.62266	7.38636	7.1889	7.19354	8.60239	8.61946	8.53263	7.93988
AHSG	0.00326809	0.34208826	5.31895219	4.77507753	5.79579407	5.17588432	6.66301857	6.96121131	6.87621894	6.75549738
ALDH1A3	0.00183073	2.91633701	9.47477586	10.0790383	9.25120426	9.63248156	7.15651354	8.84120876	7.90371804	8.0561288
ANXA2	0.00987526	0.44494842	8.19742	7.55716	8.13764	8.73795	8.91446	9.5387	9.11079	9.73938
APOLD1	0.00401784	7.20598314	10.7641727	11.8680319	10.2376323	9.51781912	6.73544791	8.01470703	9.13175638	7.50913972
C2	3.1525E-05	19.4154821	12.7529945	12.871815	13.2839544	11.8268275	8.8856567	7.06405988	8.90968942	8.49423966
C4B	0.00115237	2.74994817	11.9378609	11.8222955	12.0389152	11.2266874	10.6056506	10.4326532	10.0473907	10.3506235
CCL3L3	0.00998316	12.6461694	7.68269352	8.8183256	6.2158209	9.71923397	5.57439361	4.05321839	4.08676886	4.07917894
CCL4L2	1.04E-05	9.20389449	11.5978762	11.4136519	10.8131877	11.6962735	8.60884781	7.83085352	7.7201318	8.31047391
CLCF1	0.00359847	4.76951542	10.5256428	9.33377368	9.68999182	8.71536535	7.52957563	7.48139774	6.27183349	7.57980456
COL4A4	0.00135224	2.49753594	12.1919058	11.7701501	11.5241023	11.404309	10.4232377	10.6671093	9.81380026	10.1070536
COPS2	0.00792557	0.42881507	6.0984	6.83378	6.89997	6.91425	7.85948	7.87683	7.94789	7.94849
CP	0.00241811	2.99616713	5.80698796	5.40007396	5.5354175	5.33818918	4.30646015	3.32968881	3.40862181	3.41651403
CXCL1	7.4221E-05	86.4843126	11.686237	11.9387947	11.2808279	9.49721553	5.63286437	4.55714306	2.80740022	5.56790843
CXCL2	0.00012495	367.70035	15.0068893	13.8201615	14.3591636	12.4651913	6.24723431	5.66579877	4.88172552	5.79990993
DARS	0.00940061	0.45845435	8.23719	7.85633	7.3066	7.83945	8.91135	8.99147	8.91083	8.92652
ESPN	0.00708645	0.47468736	8.29648602	8.67334039	7.9926883	8.02194845	8.66648573	9.67202348	9.86525274	8.95570941
FGA	0.00437719	12.8132363	6.48496942	4.67359549	4.70337065	4.20977557	1.61499472	1.11357494	1.5563747	1.72366248
FGF18	0.00935827	2.38085221	6.70328291	7.65333336	6.59544475	6.55731227	5.23970752	5.50275466	5.48558436	6.43298831
FGF7	0.00739996	2.54229134	12.1623671	12.1832116	12.1907437	11.1305607	9.73457246	10.5600605	11.2887263	10.7080172
FLNA	0.00076587	3.75154573	5.87182555	6.77771993	6.1317294	5.67929733	4.27159295	3.52889417	4.38132781	4.31474657
GC	0.00356756	3.91364004	9.06839617	8.11646476	8.36148645	8.28902234	5.98313374	6.83362903	6.68717464	4.97410561
HBEGF	0.00324271	2.26897147	10.3520749	10.9191353	10.348654	9.97011992	9.23237839	9.48642214	8.86099	8.79260974
HLA-G	0.00590275	5.84427995	15.3765158	15.9942084	14.4006128	15.9280594	13.0003462	12.8258912	12.9697075	12.9126744
HP	0.00254839	18.2202734	13.6739025	12.5245569	12.4304238	11.3895733	8.53412305	8.35995836	8.84277841	8.63943997
IL6	0.00983112	3.12644152	4.22410051	3.96498279	2.78549987	4.22104595	2.67494443	2.18442782	2.37533145	2.5371227
ISYNA1	0.0065824	0.29141509	7.88827	7.06827	6.69034	6.7121	8.94954	8.8417	8.71896	8.96419
MAP2K6	0.00455522	0.45182298	5.29722036	5.62548368	5.78430258	6.22709906	6.9182892	6.45610449	7.20915294	7.36024103
MAPT	0.00537519	0.14144899	1.57334382	3.73114395	2.97726248	2.96884428	4.24416059	5.75627467	6.46324583	6.07349843
MMP7	0.00219659	4.09347148	10.1606065	9.566944	9.82729182	8.78421324	7.07419672	7.90276921	7.97790813	7.40237476
MRI1	0.00431771	0.36765449	7.33554	6.89236	6.28269	6.41489	8.06119	8.07761	8.02313	8.53786
MSLN	0.00044209	0.14342752	6.95196	6.70823	6.86536	7.003	7.88779	7.58554	7.91533	7.51336
NAMPT	0.00985356	7.12333821	10.2533392	10.2871112	10.1812931	8.28211893	7.0811292	7.04565179	6.96362138	6.81762452
NNMT	0.00186303	5.45698982	12.8814489	12.3220252	11.7445027	12.8092862	9.07053015	10.7388921	9.14951311	8.94578602
NRCAM	0.00236124	0.42474301	7.39329724	6.7680951	6.56594077	6.96481535	7.97358683	7.92348316	8.70395225	8.45748763
ORM2	0.00511354	3.82826774	6.1534025	7.19668784	5.70180137	6.04787335	3.89751055	4.20373373	5.67592806	3.23240468
PAEP	0.0083141	0.08884643	6.76843	6.6718	6.94806	7.02895	8.00595	8.37603	8.07525	7.16545
S100A12	0.00232373	3.97898962	5.61203255	6.8488888	6.52976172	6.95575669	3.73285938	5.18151433	4.16213817	3.96630919
S100A9	0.00232373	3.97898962	5.61203255	6.8488888	6.52976172	6.95575669	3.73285938	5.18151433	4.16213817	3.96630919
SAA4	0.00207474	2.05551316	6.82823229	6.39826723	6.45069781	6.66645349	6.04622935	5.42341013	4.9226849	5.48645828
SDC1	0.0075914	0.48735874	8.51797459	9.03812532	8.42085884	8.14165357	9.48961545	9.70647854	9.68265454	9.68603121
SOD2	0.00078175	40.9562282	10.5869783	9.65449752	10.1830021	8.28093542	4.88209543	4.87726698	4.11431041	4.61020239
STAT3	0.00412708	3.79616973	4.88209543	6.3085295	5.76673961	6.02454895	4.26066628	3.87347693	3.64686163	4.15932674
TARS	0.00365672	0.49813891	7.32907	7.52063	7.37836	7.76249	8.71216	8.84384	8.32002	8.13605
THBS1	0.00088828	6.28157584	11.2111301	11.2299	11.1459108	10.2206037	7.97178309	8.75060394	7.95239015	6.81845554
TNF	9.40E-05	4.50908045	8.11290726	8.01025217	8.23929127	7.85841769	5.726613	5.81210033	5.18943856	6.27183349
TP53AIP1	0.00686152	0.30677867	2.10064498	2.83702332	2.79723572	3.60309849	4.61363812	4.84750613	4.2526069	4.44317103
VAT1	0.0098171	5.68035869	8.30961818	8.04460348	9.78243274	7.36008458	6.6907511	5.14824634	4.38213199	7.15603913
WNT10A	0.0083653	2.22373592	5.95606016	5.38966095	5.24690203	5.14056709	4.95398956	4.29821536	3.30995213	4.4930245
