# Supplementary material for: Integrated analysis of mRNA and protein expression profiling in tubal endometriosis
Source: Reproduction. 2020 Mar 2;159(5):601–14. doi: 10.1530/REP-19-0587 (PMC7159149; doi:10.1530/REP-19-0587)
Supplement: Table S5. 52 Differentially expressed proteins LC-MS/MS information [file supplementary_table_5.pdf]

Table S5. 52 Differentially expressed proteins LC-MS/MS information

Protein	FDR-adjusted p-value	Fold Change relative to control	S129922_TEM	S302873_TEM	S30589_TEM	S30863_TEM	S30353_NC	S30504_NC	S30622_NC	S306515_NC
A1BG	0.00919397	2.08500017	8.35491	8.77225	8.26081	8.49668	7.20962	7.28237	7.10442	8.04805
C2	0.00650354	5.67648538	12.3849129	12.8755425	11.7658302	11.7560978	10.4748677	9.66946809	8.3693694	10.2486865
C4B	0.00893826	6.17940019	13.6936945	13.5008415	11.9816996	13.1519892	10.6278418	10.9530104	8.90159981	11.3359055
CAP1	0.00303174	2.16155568	8.5369	8.36996	8.67264	8.25005	7.80634	7.35434	6.98425	7.23634
CCL3L3	0.00998316	12.6461694	7.68269352	8.8183256	6.2158209	9.71923397	5.57439361	4.05321839	4.08676886	4.07917894
CCL4L2	2.8247E-05	9.59757923	11.5978762	11.4136519	10.8131877	11.6962735	8.60884781	7.83085352	7.7201318	8.31047391
CFB	0.00657697	2.8715158	8.96958	9.6031	9.48866	8.72976	8.36177	7.19504	7.19659	7.95045
COL4A4	0.00917772	3.63361561	5.62754591	6.51084788	6.22709906	7.26905379	4.60391958	4.39381342	4.79529644	4.39589395
CP	0.00935563	14.3898292	11.1932322	11.2150262	10.4071762	10.1969153	8.56471752	6.82271859	4.99459335	7.24241011
CXCL1	0.00033147	88.0004987	11.686237	11.9387947	11.2808279	9.49721553	5.63286437	4.55714306	2.80740022	5.56790843
CXCL2	0.00109296	182.14281	13.1170869	11.6675799	12.5705218	9.91265424	4.59573021	4.50813882	3.78979408	4.33847479
FGA	0.00426989	11.4381044	6.48496942	4.67359549	4.70337065	4.20977557	1.61499472	1.11357494	1.5563747	1.72366248
FGF18	0.00678236	7.37642609	9.40745577	8.09045537	10.0066116	7.97450216	6.2521927	6.29937059	4.51995524	6.8758184
FGF7	0.00719728	4.07959378	8.1669885	9.25120426	7.95170363	7.90986914	5.86204546	7.31303082	6.43838317	5.55260405
FLNA	0.0059687	2.03296095	9.35904	8.98349	9.80796	9.48234	8.11916	8.56651	8.05801	8.79482
GC	0.00691915	2.21040388	9.49575	9.3104	9.4952	9.38353	8.70409	8.13389	7.83722	8.43244
HBEGF	0.00965723	8.03520809	7.58747243	9.9198342	7.11137599	7.72502486	4.46243916	4.14034076	5.90867507	5.80691093
HLA-G	0.00684797	3.68482898	4.65550296	5.16588265	4.13824587	5.62298916	2.28729844	2.97688897	2.87198193	3.92006064
HNRNPA2B1	0.00410908	2.29691107	8.92844	8.7361	8.13194	8.55368	7.38487	7.89832	6.91771	7.35048
HP	0.00713682	27.8694942	10.2932421	13.984608	10.3499983	10.5634241	5.83501107	7.69076654	5.97456573	6.48846961
IGLL5	0.00972356	2.50319815	7.99706	8.49766	8.6621	8.74108	6.9191	7.65546	6.47783	7.55042
IL6	0.00984178	34.8412574	8.83791594	10.7190878	7.39390701	6.7230179	3.29478822	3.08326203	3.28962751	3.51535178
MMP7	0.00683678	24.6815116	14.6532148	13.4611436	14.4773461	14.2893683	11.0387557	10.9035261	8.16733193	8.27002362
ORM2	0.00281776	2.00264611	7.31983	8.07181	7.61927	7.75035	6.90696	6.73939	6.78884	6.31844
S100A9	0.00443157	39.0022379	13.7652	10.66654	10.2903	10.71072	6.85066	5.98613	5.50969	5.94434
SAA2-SAA4	0.00710095	2.42775023	8.87347	8.61639	8.74221	8.45327	8.04012	7.34914	6.96846	7.20914
SAA4	0.00710026	29.2879385	7.79207308	10.3626942	7.78827895	5.92866558	3.56738411	2.42608036	1.34716625	5.04214207
SERPINA1	0.00566953	2.63430976	10.2167	10.5686	10.3469	9.39952	8.33298	8.97547	8.69073	8.94284
SNCA	0.00622196	3.86522527	8.20672	8.68673	8.55149	7.25853	6.08695	6.25188	6.06344	6.49899
TNF	0.00081308	4.94243076	8.11290726	8.01025217	8.23929127	7.85841769	5.726613	5.81210033	5.18943856	6.27183348
VAT1	0.00630013	2.15137646	8.57586	8.41601	8.21611	7.82974	7.77279	6.96091	6.92761	6.95537
VCL	0.00060262	2.42762823	9.15512	9.22766	8.81525	8.85022	7.64926	7.92927	7.99621	7.35532
WNT10A	0.00699026	2.97588237	7.91250125	6.46386442	7.13158659	7.44988231	5.78503786	5.03556404	5.90988501	5.93407767
AGL	0.00307397	0.37586005	7.5548	7.18304	6.9997	7.40161	8.06352	8.99702	8.72881	8.99673
AHSG	0.00326809	0.34208826	5.31895219	4.77507753	5.79579407	5.17588432	6.66301857	6.96121131	6.87621894	6.75549738
ANXA2	0.00987526	0.44494842	8.19742	7.55716	8.13764	8.73795	8.91446	9.5387	9.11079	9.73938
EZR	0.00603152	0.3822491	8.69011	8.44105	8.00612	7.6835	9.54301	9.55495	9.48841	9.78407
HSPB1	0.00265115	0.49461744	8.55706	8.83749	8.09065	8.38653	9.45242	9.43111	9.22032	9.83034
HSPH1	0.00843181	0.33303253	7.68508	7.567	7.19449	7.64226	9.40848	8.24561	9.20344	9.57636
MAP2K6	0.00404865	0.41974339	5.29722036	5.62548368	5.78430258	6.22709906	6.9182892	6.45610449	7.20915294	7.36024103
MAPT	0.00537519	0.14144899	1.57334382	3.73114395	2.97726248	2.96884428	4.24416059	5.75627467	6.46324583	6.07349843
MRI1	0.00431771	0.36765449	7.33554	6.89236	6.28269	6.41489	8.06119	8.07761	8.02313	8.53786
NRCAM	0.00209036	0.39458545	7.39329724	6.7680951	6.56594077	6.96481535	7.97358683	7.92348316	8.70395225	8.45748763
PAEP	2.7937E-05	0.40574353	6.76843	6.6718	6.94806	7.02895	8.00595	8.37603	8.07525	8.16545
PFKP	0.00476322	0.42072079	7.16501	7.46636	7.45154	7.2944	8.98778	8.76525	8.55496	8.06558
PSMA7	0.00983617	0.29412677	7.53232	7.40851	6.77034	7.53747	9.1614	8.18367	9.95962	9.00591
S100A11	1.0391E-05	0.33344541	8.03599	7.74406	7.79628	8.02346	9.22686	9.59344	9.57356	9.54384
SDC1	0.00720338	0.46279916	8.51797459	9.03812532	8.42085884	8.14165357	9.48961545	9.70647854	9.68265454	9.68603121
SHMT1	0.00026804	0.3466526	7.42339	7.1543	7.20585	7.31725	8.4334	8.87157	8.99409	8.91548
TP53AIP1	0.00686152	0.30677867	2.10064498	2.83702332	2.79723572	3.60309849	4.61363812	4.84750613	4.2526069	4.44317103
UGDH	0.00334336	0.29241262	8.46875	7.33983	7.24509	7.52648	9.61463	9.35626	9.01899	9.68596
MSLN	0.00044209	0.14342752	6.95196	6.70823	6.86536	7.003	7.88779	7.58554	7.91533	7.51336
