# Supplementary material for: Integrated analysis of mRNA and protein expression profiling in tubal endometriosis
Source: Reproduction. 2020 Mar 2;159(5):601–14. doi: 10.1530/REP-19-0587 (PMC7159149; doi:10.1530/REP-19-0587)
Supplement: Table S6. Details for the annotation used for protein identifiers. [file supplementary_table_6.pdf]

Table S6. Details for the annotation used for protein identifiers.

Protein IDs	Protein names	Gene names	Fasta headers
P04217; P04217-2; M0R009; CON-Q2KJF1	Alpha-1B-glycoprotein	*A1BG*	Alpha-1B-glycoprotein; Isoform 2 of Alpha-1B-glycoprotein; Alpha-1B-glycoprotein (Fragment)
P35573; P35573-2; P35573-3	Glycogen debranching enzyme; 4-alpha-glucanotransferase; Amylo-alpha-1,6-glucosidase	*AGL*	Glycogen debranching enzyme; Isoform 5 of Glycogen debranching enzyme; Isoform 6 of Glycogen debranching enzyme
P02765; C9JV77	Alpha-2-HS-glycoprotein; Alpha-2-HS-glycoprotein chain A; Alpha-2-HS-glycoprotein chain B	*AHSG*	Alpha-2-HS-glycoprotein; Alpha-2-HS-glycoprotein
P07355; H0YN42; P07355-2; H0YMD0; H0YMU9; H0YMM1; H0YKS4; A6NMY6; H0YKZ7; H0YLV6; H0YMT9; H0YKX9; H0YKL9; H0YMW4; H0YKV8; H0YM50; H0YMD9; H0YNP5; H0YNB8; H0YKN4; H0YN28; H0YL33; H0YNA0; H0YLE2; H0YN52	Annexin A2; Annexin; Putative annexin A2-like protein	*ANXA2; ANXA2P2*	Annexin A2; Annexin (Fragment); Isoform 2 of Annexin A2; Annexin (Fragment); Annexin; Annexin (Fragment); Annexin (Fragment); Putative annexin A2-like protein; Annexin (Fragment); Annexin; Annexin (Fragment); Annexin (Fragment); Annexin (Fragment); Annexin (Fragment);
P06681; B4DQI1; A0A0G2JL69; P06681-3; A0A0G2JIE7; H0Y3H6; P06681-2; E9PDZ0; A0A0G2JK28; H0Y868	Complement C2; Complement C2b fragment; Complement C2a fragment	*C2*	Complement C2; Complement C2; Complement C2; Isoform 3 of Complement C2; Complement C2; Complement C2 (Fragment); Isoform 2 of Complement C2; Complement C2 (Fragment); Complement C2 (Fragment)
A0A140TA29; A0A0G2JL54; F5GXS0	Complement C4-B	*C4B*	Complement C4-B; Complement C4-B; Complement C4-B
Q01518-2; Q01518; Q5T0R7; Q5T0R6; Q5T0R5; Q5T0R4; Q5T0R3; Q5T0R2; Q5T0R1; Q5T0R9; Q5T0R8; Q5T0S3	Adenylyl cyclase-associated protein 1; Adenylyl cyclase-associated protein	*CAP1*	Isoform 2 of Adenylyl cyclase-associated protein 1; Adenylyl cyclase-associated protein 1; Adenylyl cyclase-associated protein (Fragment); Adenylyl cyclase-associated protein (Fragment); Adenylyl cyclase-associated protein (Fragment); Adenylyl cyclase-associate
Q8NHW4	C-C Motif Chemokine Ligand 4 Like 2	*CCL4L2*	C-C Motif Chemokine Ligand 4 Like 2
P16619	C-C Motif Chemokine Ligand 3 Like 3	*CCL3L3*	C-C Motif Chemokine Ligand 3 Like 3
B4E1Z4; E7ETN3; P00751; A0A0G2JH38; H7C5H1; P00751-2; C9JRT3; A0A0G2JHM4; A0A0G2JJ82; H7C526; F8WCJ9; A0A0G2JJM0; Q5ST52; F2Z3N2; Q8N6L6	Complement factor B; Complement factor B Ba fragment; Complement factor B Bb fragment	*CFB*	Uncharacterized protein; Uncharacterized protein; Complement factor B
P53420	Collagen Type IV Alpha 4 Chain	*COL4A4*	Collagen Type IV Alpha 4 Chain
P00450; E9PFZ2; H7C5R1; CON__ENSEMBL:ENSBTAP00000031900; D6RE86; H7C5N5	Ceruloplasmin	*CP*	Ceruloplasmin; Ceruloplasmin; Ceruloplasmin (Fragment)
P09341	Growth-regulated alpha protein	*CXCL1*	Growth-regulated alpha protein
P19875	C-X-C motif chemokine 2	*CXCL2*	C-X-C motif chemokine 2
E7EQR4; P15311; E9PQ82	Ezrin	*EZR*	Ezrin; Ezrin
P02671-2; P02671; A0A087WUA0	Fibrinogen alpha chain; Fibrinopeptide A; Fibrinogen alpha chain	*FGA*	Isoform 2 of Fibrinogen alpha chain; Fibrinogen alpha chain
O76093	Fibroblast growth factor 18	*FGF18*	Fibroblast growth factor 18
P21781	Fibroblast growth factor 7	*FGF7*	Fibroblast growth factor 7
P21333-2; P21333; Q5HY54;	Filamin-A	*FLNA*	Isoform 2 of Filamin-A; Filamin-A;

Q60FE5; A0A087WWY3; F8WE98; H0Y5F3; H0Y5C6; H7C2E7			Filamin-A; Filamin A; Filamin-A
D6RF35; P02774; P02774-3 ; P02774-2; D6RBJ7; D6RF20; CON__Q3MHN5; CON__ENSEMBL:ENSBTAP00000018229	Vitamin D-binding protein	*GC*	Vitamin D-binding protein; Vitamin D-binding protein; Isoform 3 of Vitamin D-binding protein; I soform 2 of Vitamin D-binding protein
Q99075	Proheparin-binding EGF-like growth factor	*HBEGF*	Proheparin-binding EGF-like growth factor
P17693	HLA class I histocompatibility antigen, alpha chain G	*HLA-G*	HLA class I histocompatibility antigen, alpha chain G
P22626; P22626-2; A0A087WUI2	Heterogeneous nuclear ribonucleoproteins A2/B1	*HNRNPA2B1*	Heterogeneous nuclear ribonucleoproteins A2/B1; Isoform A2 of Heterogeneous nuclear ribonucleoproteins A2/B1; Heterogeneous nuclear ribonucleoproteins A2/B1
P00738; A0A0C4DGL8; J3QR68; H0Y300; P00738-2; J3QLC9; A0A087WU08; H3BS21; J3KRH2; J3KTC3; J3KSV1; J3QQI8; H3BMJ7	Haptoglobin; Haptoglobin alpha chain; Haptoglobin beta chain	*HP*	Haptoglobin; Haptoglobin; Haptoglobin (Fragment); Haptoglobin; Isoform 2 of Haptoglobin; Haptoglobin (Fragment); Haptoglobin;Haptoglobin (Fragment)
P04792; F8WE04; C9J3N8	Heat shock protein beta-1	*HSPB1*	Heat shock protein beta-1; Heat shock protein beta-1
Q92598-2; Q92598-3; Q92598; Q92598-4; A0A0A0MSM0; R4GN69; Q5TBM3	Heat shock protein 105 kDa	*HSPH1*	Isoform Beta of Heat shock protein 105 kDa; Isoform 3 of Heat shock protein 105 kDa; Heat shock protein 105 kDa; Isoform 4 of Heat shock protein 105 kDa; Heat shock protein 105 kDa
A0A0B4J231; B9A064; A0A075B6K8; P0CG04	Immunoglobulin lambda-like polypeptide 5; Ig lambda-1 chain C regions	*IGLL5; IGLC1*	Immunoglobulin lambda-like polypeptide 5; Immunoglobulin lambda-like polypeptide 5; Ig lambda-1 chain C regions (Fragment); Ig lambda-1 chain C regions
P05231	Interleukin-6	*IL6*	Interleukin-6
P52564	Mitogen-Activated Protein Kinase Kinase 6	*MAP2K6*	Mitogen-Activated Protein Kinase Kinase 6
P09237	Matrix Metallopeptidase 7	*MMP7*	Matrix Metallopeptidase 7
P10636	Microtubule-associated protein tau	*MAPT*	Macrophage migration inhibitory factor
Q9BV20-2; Q9BV20	Methylthioribose-1-phosphate isomerase	*MRI1*	Isoform 2 of Methylthioribose-1-phosphate isomerase; Methylthioribose-1-phosphate isomerase
H3BMA1; H3BUX1; Q13421-4; Q13421-3; Q13421-2; Q13421; H3BR90	Mesothelin; Megakaryocyte-potentiating factor; Mesothelin, cleaved form	*MSLN*	Mesothelin (Fragment); Mesothelin (Fragment); Isoform 4 of Mesothelin; Isoform 2 of Mesothelin; Isoform 3 of Mesothelin; Mesothelin
Q92823	Neuronal cell adhesion molecule	*NRCAM*	Neuronal cell adhesion molecule
P19652	Alpha-1-acid glycoprotein 2	*ORM2*	Alpha-1-acid glycoprotein 2
H0Y6A4; H0Y4U4; Q5T6T6; A6XNE0; P09466-2; P09466; P09466-3; H0Y530; F2Z349	Glycodelin	*PAEP*	Glycodelin (Fragment); Glycodelin (Fragment); Glycodelin (Fragment); Glycodelin; Isoform 2 of Glycodelin; Glycodelin; Isoform 3 of Glycodelin
Q01813; Q01813-2; Q5VSR5; B1APP6; H0Y3Y3; B1APP8; H0Y757; V9GYV7; V9GY25	ATP-dependent 6-phosphofructokinase, platelet type	*PFKP*	ATP-dependent 6-phosphofructokinase, platelet type; Isoform 2 of ATP-dependent 6-phosphofructokinase, platelet type
O14818; O14818-2; H0Y586; Q8TAA3-2; Q8TAA3-5; Q8TAA3; F5GY34; A0A087WYS6; O14818-4	Proteasome subunit alpha type-7; Proteasome subunit alpha type-7-like	*PSMA7*	Proteasome subunit alpha type-7;I soform 2 of Proteasome subunit alpha type-7; Proteasome subunit alpha type-7 (Fragment); Isoform 2 of Proteasome subunit alpha type-7-like;Isoform 3 of Proteasome subunit alpha type-7-like; Proteasome subunit alpha type-7-like
P31949	Protein S100-A11; Protein S100-A11, N-terminally processed	*S100A11*	Protein S100-A11
P06702	Protein S100-A9	*S100A9*	Protein S100-A9

A0A096LPE2; P35542	Serum amyloid A-4 protein	*SAA2-SAA4;SAA4*	Protein SAA2-SAA4; Serum amyloid A-4 protein
P35542	Serum amyloid A-4 protein	*SAA4*	Serum amyloid A-4 protein
P01009; A0A024R6I7;P01009-2;A0A0G2JRN3;P01009-3;G3V2B9;G3V544;G3V5R8;G3V387;A0A0B4J278;A0A0G2JPK4;P20848	Alpha-1-antitrypsin; Short peptide from AAT	*SERPINA1*	Alpha-1-antitrypsin; Alpha-1-antitrypsin; Isoform 2 of Alpha-1-antitrypsin; Alpha-1-antitrypsin; Isoform 3 of Alpha-1-antitrypsin
P18827	Syndecan-1	*SDC1*	Syndecan-1
P34896-4; P34896-2;P34896; P34896-3;G3V2D2; G3V540; G3V5L0; G3V2Y4; G3V4W5; P34897-3; P34897-2; P34897	Serine hydroxymethyltransferase, cytosolic	*SHMT1*	Isoform 4 of Serine hydroxymethyltransferase, cytosolic; Isoform 2 of Serine hydroxymethyltransferase, cytosolic; Serine hydroxymethyltransferase, cytosolic; Isoform 3 of Serine hydroxymethyltransferase, cytosolic
E7EPV7; P37840-2; P37840; H6UYS7; P37840-3; D6RA31	Alpha-synuclein	*SNCA*	Alpha-synuclein; Isoform 2-4 of Alpha-synuclein; Alpha-synuclein; Alpha-synuclein; Isoform 2-5 of Alpha-synuclein
P01375	Tumor necrosis factor	*TNF*	Tumor necrosis factor
Q9HCN2	p53-regulated apoptosis-inducing protein 1	*TP53AIP1*	p53-regulated apoptosis-inducing protein 1
O60701; O60701-2;O60701-3; E7ER95; E7ER83; E7ETF4; E7EV97; D6RHF4; E9PBD2	UDP-glucose 6-dehydrogenase	*UGDH*	UDP-glucose 6-dehydrogenase; Isoform 2 of UDP-glucose 6-dehydrogenase; Isoform 3 of UDP-glucose 6-dehydrogenase
Q99536; Q99536-3; Q99536-2; K7ESA3; K7ERT7; K7EJM4; K7ENX2; K7EM19; K7ER81	Synaptic vesicle membrane protein VAT-1 homolog	*VAT1*	Synaptic vesicle membrane protein VAT-1 homolog; Isoform 3 of Synaptic vesicle membrane protein VAT-1 homolog; Isoform 2 of Synaptic vesicle membrane protein VAT-1 homolog
P18206-2; P18206; Q5JQ13; P18206-3	Vinculin	*VCL*	Isoform 1 of Vinculin; Vinculin; Vinculin (Fragment)
Q9GZT5	Wnt Family Member 10A	*WNT10A*	Wnt Family Member 10A
