# Supplementary figures and images for: Integrated analysis of mRNA and protein expression profiling in tubal endometriosis
Source: Reproduction. 2020 Mar 2;159(5):601–14. doi: 10.1530/REP-19-0587 (PMC7159149; doi:10.1530/REP-19-0587)

**Fig S1**

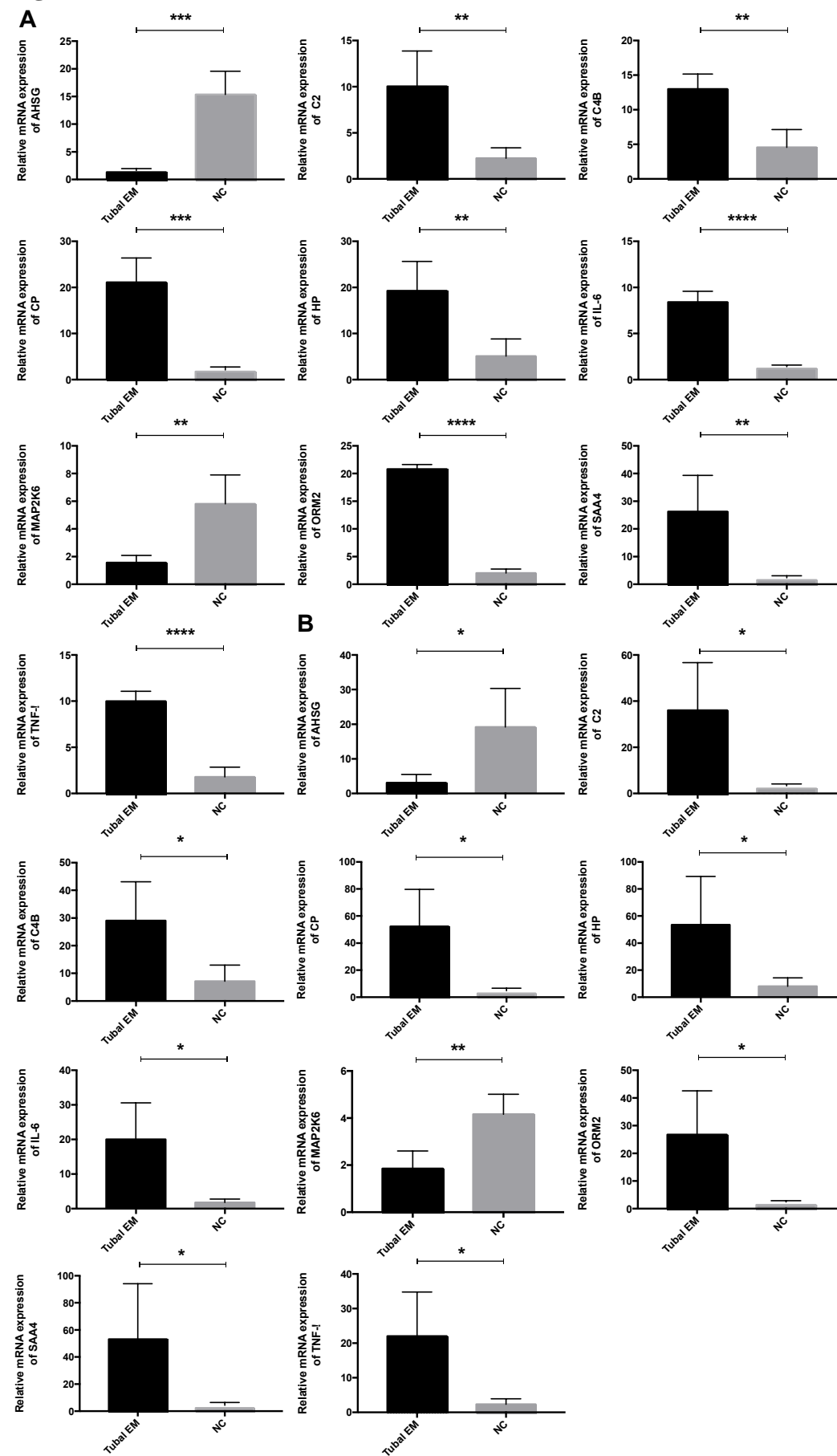

Supplement: Fig S1 [file supplementary_figure_1.pdf]
